# Safe and efficient *in vivo* hematopoietic stem cell transduction in nonhuman primates using HDAd5/35++ vectors

**DOI:** 10.1016/j.omtm.2021.12.003

**Published:** 2021-12-06

**Authors:** Chang Li, Hongjie Wang, Sucheol Gil, Audrey Germond, Connie Fountain, Audrey Baldessari, Jiho Kim, Zhinan Liu, Aphrodite Georgakopoulou, Stefan Radtke, Tamás Raskó, Amit Pande, Christina Chiang, Eli Chin, Evangelia Yannaki, Zsuzsanna Izsvák, Thalia Papayannopoulou, Hans-Peter Kiem, André Lieber

**Affiliations:** 1 University of Washington, Department of Medicine, Division of Medical Genetics, Box 357720, Seattle, WA 98195, USA; 2 Washington National Primate Research Center, Seattle, WA 98195, USA; 3 Gene and Cell Therapy Center, Hematology Department, George Papanicolaou Hospital, Thessaloniki 57010, Greece; 4 Stem and Gene Therapy Program, Fred Hutchinson Cancer Research Center, Seattle, WA 98109, USA; 5 AG "Mobile DNA Lab" Max Delbrück Center for Molecular Medicine, 13125 Berlin-Buch, Germany; 6 University of Washington, Department of Medicine, Division of Hematology, Box 357720, Seattle, WA 98195, USA; 7 University of Washington, Department of Medicine, Division of Medical Oncology, Box 357720, Seattle, WA 98195, USA; 8 University of Washington, Department of Pathology, Seattle, WA 98195, USA

**Keywords:** *in vivo*, hematopoietic stem cells, adenovirus vector, nonhuman primates, hemoglobinopathies, gamma globin

## Abstract

We tested a new *in vivo* hematopoietic stem cell (HSC) transduction/selection approach in rhesus macaques using HSC-tropic, integrating, helper-dependent adenovirus vectors (HDAd5/35++) designed for the expression of human γ-globin in red blood cells (RBCs) to treat hemoglobinopathies. We show that HDAd5/35++ vectors preferentially transduce HSCs *in vivo* after intravenous injection into granulocyte colony-stimulating factor (G-CSF)/AMD3100-mobilized animals and that transduced cells return to the bone marrow and spleen. The approach was well tolerated, and the activation of proinflammatory cytokines that are usually associated with intravenous adenovirus vector injection was successfully blunted by pre-treatment with dexamethasone in combination with interleukin (IL)-1 and IL-6 receptor blockers. Using our MGMT^P140K^-based *in vivo* selection approach, γ-globin^+^ RBCs increased in all animals with levels up to 90%. After selection, the percentage of γ-globin^+^ RBCs declined, most likely due to an immune response against human transgene products. Our biodistribution data indicate that γ-globin^+^ RBCs in the periphery were mostly derived from mobilized HSCs that homed to the spleen. Integration site analysis revealed a polyclonal pattern and no genotoxicity related to transgene integrations. This is the first proof-of-concept study in nonhuman primates to show that *in vivo* HSC gene therapy could be feasible in humans without the need for high-dose chemotherapy conditioning and HSC transplantation.

## Graphical abstract

**Figure:**
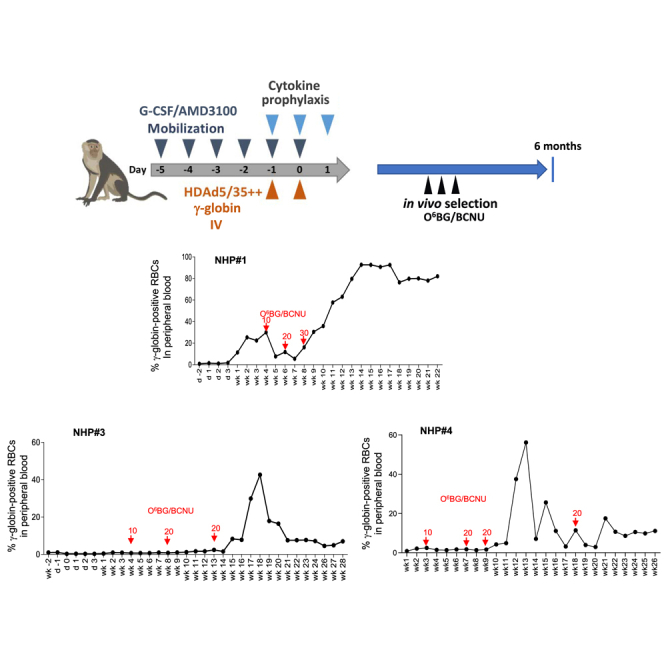

## Teaser

We have developed an *in vivo* hematopoietic stem cell (HSC) gene therapy approach that involves HSC mobilization and intravenous injection of HSC-tropic, integrating HDAd5/35++ vectors. Our study indicates that this approach is safe and effective in rhesus macaques and could be feasible in humans.

## Introduction

Autologous hematopoietic stem cell (HSC) gene therapy for hemoglobinopathies has shown promising effective cures.^1^, ^2^, ^3^, ^4^ Despite the encouraging clinical results, current *ex vivo* HSC gene therapy protocols have multiple shortcomings throughout the process: (1) harvesting HSCs by leukapheresis or bone marrow aspiration (invasive procedure); (2) myeloablation by chemotherapy (high-dose-chemotherapy-related side effects, infectious disease complications, conditioning-associated genotoxicity), (3) *in vitro* HSC culture and transplantation (loss of HSC pluripotency during extended *ex vivo* culture, need for specialized facility/staff); and (4) the cost of the approach. Because of the cost and technical complexity, it is unlikely that *ex vivo* protocols will be widely applicable, specifically in developing countries where the greatest demand for hemoglobinopathy therapy lies.

We are working on an approach for the transduction of HSCs *in vivo* using helper-dependent adenovirus vectors systems (HDAd5/35++). So far, we have published safety and efficacy data obtained in mice.^5^, ^6^, ^7^, ^8^, ^9^, ^10^, ^11^, ^12^, ^13^, ^14^, ^15^, ^16^

Our approach involves the mobilization of HSCs from the bone marrow by a granulocyte colony-stimulating factor (G-CSF) and the short-acting CXCR4 antagonist Plerixafor/AMD3100. While mobilized HSCs circulate at high numbers in the periphery, HDAd5/35++ vectors are injected intravenously.^17^ The mobilization of HSCs is critical for *in vivo* transduction because in the bone marrow they are surrounded by extracellular stroma proteins^18^ and are not accessible to gene transfer vectors.^12^ HDAd5/35++ vectors are easy to manufacture at high yields, can carry a payload of 35 kb, and can efficiently transduce primitive, quiescent HSCs through CD46.^12^ The vectors’ affinity to CD46 has been increased^19^ to allow for *in vivo* HSC transduction without significant vector uptake by hepatocytes.^20^^,^^21^ The random integration of HDAd5/35++ vectors is mediated by an activity-enhanced *Sleeping Beauty* transposase (SB100x).^22^ To expand transduced HSCs, we currently use an *in vivo* selection mechanism based on a mutant O^6^-methylguanine-DNA methyltransferase (mgmt^P140K^) gene that confers resistance to O^6^-BG/BCNU (O^6^-benzylguanine/carmustine).^16^^,^^23^^,^^24^ After *in vivo* transduction and selection with three low doses of O^6^-BG/BCNU administered intraperitoneally at an interval of 2 weeks, transgene marking in peripheral blood mononuclear cells (PBMCs) was usually increased to >90%.^16^ We have shown that our approach resulted in a phenotypic correction in mouse disease models of thalassemia intermedia,^14^ sickle cell disease, and murine hemophilia A^15^ and in the reversion of spontaneous cancer.^25^

The biodistribution and function of CD46 in mice and humans are different.^26^ In humans, CD46 is present on all nucleated cells, while the expression of the mouse CD46 orthologue is restricted to the testis. For our *in vivo* HSC transduction studies, we therefore used human CD46 transgenic mice. These mice carry 400 kb of the human CD46 locus^27^ and express the protein in a pattern similar to humans.^28^ It remains, however, unclear whether human CD46 is able to trigger intracellular signaling in this heterologous transgenic mouse model. Furthermore, innate and adaptive immune responses initiated by intravenous HDAd5/35++ injection might not be adequately reflected in the mouse model. The physiological similarities of the human and macaque hematopoietic systems make rhesus macaques (*Macaca mulatta*) a better model for a potential clinical translation of our *in vivo* HSC gene therapy approach. The expression pattern of CD46 in rhesus macaques is similar to humans, with a notable exception that CD46 is found on red blood cells (RBCs).^29^^,^^30^ Intravenous injection of CD46-targeting Ad5/35^31^ and Ad35 vectors^32^ into nonhuman primates (NHPs) did not result in an efficient transduction of normal tissues, most likely because of the low CD46 density on differentiated cells^33^ and inaccessibility due to localization in epithelial junctions.^34^

Here, we tested our *in vivo* HSC transduction/selection approach in rhesus macaques.

## Results

### Development of an *in vivo* HSC transduction approach for NHPs

#### HSC targeting through CD46 with HDAd5/35++ vectors

We have recently reported that CD46 is expressed at a higher level on human CD34^+^ cells compared to other mononuclear cells in the bone marrow and peripheral blood,^12^ suggesting that CD46 also has (a yet unknown) function in HSCs. In Figure S1A, we show uniform high expression of CD46 on primitive rhesus HSCs (CD34^+^/CD45RA^–^/CD90^+^ cells).^35^ *In vitro* transduction of human and rhesus CD34+ cells with a GFP-expressing HDAd5/35++ vector yielded higher percentages of GFP-positive cells (50%–65%) in CD34^+^/CD45RA^–^/CD90^+^ cells than in the pool of CD34^+^ cells, most likely due to a higher CD46 density (Figures S1B–S1D). This study suggests that HDAd5/35++ vectors are suitable for *in vivo* HSC transduction in rhesus macaques.

**Figure 1:**
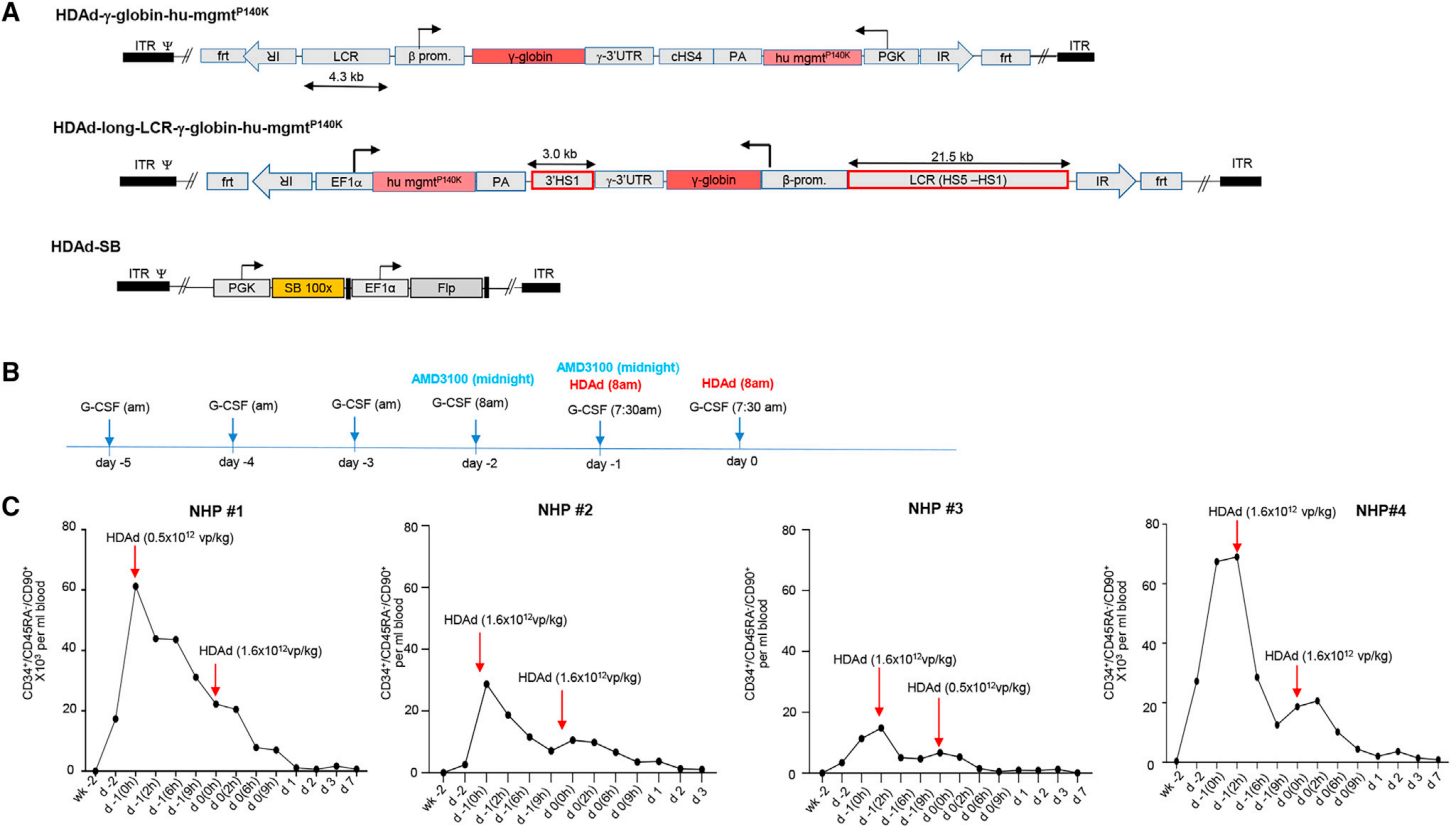
Injection of HDAd5/35++ vectors for γ-globin gene addition into mobilized rhesus macaques (A) Vector structure. γ-globin gene addition is achieved through the SB100x transposase system consisting of a transposon vector with inverted repeat (IR) sequences and *frt* sites flanking the expression cassette and a second vector (HDAd-SB) that provides the SB100x and Flpe recombinase *in trans*.^22^ Animals received the SB100x/Flpe-expressing HDAd-SB vector together with a γ-globin-expressing transposon vector (1:1 ratio). HDAd-γ-globin-mgmt^P140K^: The transposon cassette for random integration consists of a mini β-globin LCR/promoter for erythroid-specific expression of human γ-globin (HBG1, 76-Ile variant). The γ-globin 3′ UTR serves for mRNA stabilization in erythroid cells. The γ-globin expression unit is separated by a chicken globin HS4 insulator from a cassette for human mgmt^P140K^ expression from a ubiquitously active PGK promoter. In HDAd-long-γ-globin-hu-mgmt^P140K^, the human γ-globin gene is under the control of a 21.5 kb β-globin LCR (chr11: 5292319–5270789), a 1.6 kb β-globin promoter (chr11: 5228631–5227023), and a 3′ HS1 region (chr11: 5206867–5203839) also derived from the β-globin locus.^13^ (B) Timing of G-CSF, AMD3100, and HDAd5/35++ vector injection. (C) Numbers of primitive (CD34^+^/CD45RA^–^/CD90^+^) HSCs in peripheral blood. HDAd5/35++ vectors were injected at the two peaks of mobilization. Vector dosing in NHP#1 was conservative (0.5 → 1.6 × 10^12^ vp/kg). The other animals were dosed two times with 1.6 × 10^12^ vp/kg except NHP#3. The low second HDAd5/35++ dose in NHP#3 was based on a worrisome neutrophil count received shortly before the second HDAd5/35++ injection. Later, this count was found to be erroneous. The time of HDAd5/35++ injection is indicated as “0 h” correspondingly at days −1 and 0. After this timepoint, blood samples were analyzed at 2, 6, and 9 h on days −1 and 0 and then on days 1, 2, 3, and 7.

Our goal was to test HDAd5/35++ vectors for gene therapy of hemoglobinopathies based on promising studies performed in CD46-transgenic mouse models for thalassemia and sickle cell disease.^10^^,^^14^^,^^36^ Four rhesus macaques were treated successively (Table 1). Based on the data from the n = 1 studies, we modified the experimental design for the next animal (Table 2). All vectors tested contained a cassette for random chromosomal integration of the human γ-globin gene (HBG1) and the human mgmt^P140K^ gene. We are using two vectors to achieve transgene integration: (1) a transposon vector containing the therapeutic cargo sequence flanked by SB100x-inverted repeats and *frt* sites, and (ii) the transposase vector HDAd-SB to provide SB100x and Flpe (for transposon circularization) *in trans*^22^^,^^37^ (Figure 1A). NHPs#1 and #3 received a vector containing the human γ-globin gene under the control of the 4.3 kb “short” β-globin locus control region (LCR) for erythroid-specific expression. NHP#4 received a vector containing a ∼25 kb long β-globin LCR to maximize γ-globin expression levels.^36^

**Table 1:** Information on animal weight, age, source, and study duration

Animal	ID	Age (years)	Weight (kg)	Gender	Source	Study duration
NHP#1	A17284	5.6	11.5	M	ONPRC	08/12/19–01/06/20
NHP#2	A19237	5.5	9.0	M	ONPRC	01/26/20–01/31/20
NHP#3	A19238	4.4	6.0	M	ONPRC	03/01/20–09/08/20
NHP#4	A20141	8.0	5.8	F	ONPRC	01/11/21–07/21/21
NHP-Co	A20143	5.2	5.7	F	ONPRC	05/01/21–05/04/21

ONPRC, Oregon National Primate Research Center.

**Table 2:** Information on HDAd vectors, doses, cytokine prophylaxis, immunosuppression, and *in vivo* selection

Animal	Vector doses (vp/kg)	Expression cassettes	Cytokine prophylaxis	Immuno-suppression	*In vivo* selection O^6^BG/BCNU (mg/m^2^)
NHP#1	0.5/1.6 × 10^12^	γ-globin, hu-mgmt^P140K^, SB100x, Flpe	Dex, tocilizumab	tacrolimus (s.c.), MMF (PO), sirolimus (i.m.)	week 4: 120/10week 6: 120/20week 8: 120/30
NHP#2	1.6/1.6× 10^12^	γ-globin, hu-mgmt^P140K^, targeted integration	Dex, tocilizumab, anakinra (i.v.)	tacrolimus (s.c.), MMF (gastric catheter)	none
NHP#3	1.6/0.5 × 10^12^	γ-globin, hu-mgmt^P140K^, SB100x, Flpe	Dex, tocilizumab, anakinra (i.v.)	tacrolimus (s.c.), MMF(PO), abatacept (s.c., weeks 21–24)	week 4: 120/10week 8: 120/20week 13: 120/20
NHP#4	1.6/1.6 × 10^12^	γ-globin, long-LCR hu-mgmt^P140K^, SB100x, Flpe	Dex, tocilizumab, anakinra (s.c.)	tacrolimus (s.c.), MMF (PO), abatacept (s.c., day 0 to week 3)	week 3: 120/10week 7: 120/20week 9: 120/20week 18: 120/20
NHP-Co	1.6/1.5 × 10^12^	γ-globin, long-LCR hu-mgmt^P140K^, SB100x, Flpe	Dex, tocilizumab, anakinra (s.c.)	none	none

NHP#2 was injected with a HDAd5/35++ vector designed for targeted integration of the γ-globin cassette into a safe genomic harbor (PMID: 31494053). However, NHP#2 had to be euthanized on day 3 after HDAd injection due to an erroneous tacrolimus overdose (given through a gastric catheter). NHP-Co was added to assess the effect of mobilization on vector biodistribution. SB100x, activity-enhanced *Sleeping Beauty* transposase; Flpe, activity-enhanced Flp recombinase; LCR, β-globin locus control region; s.c., subcutaneous; PO, per os – oral; i.m., intramuscular.

NHP#2 was injected with a HDAd5/35++ vector designed for the targeted integration of the γ-globin cassette into a safe genomic harbor.^8^ However, this animal had to be euthanized on day 3 after HDAd injection due to an erroneous tacrolimus overdose (given through a gastric catheter). Our long-term data are therefore only from NHPs#1, #3, and #4.

#### HSC mobilization and timing of HDAd5/35++ injection

CD46 on rhesus RBCs can sequester HDAd5/35++ virions, thus serving as a vector trap following intravenous administration. This is supported by an *in vitro* study (Figure S2). In an attempt to mitigate this issue by increasing the vector dose, we established an HSC mobilization regimen that would involve two waves of mobilization. At the peak of each, we would then intravenously inject the HDAd5/35++ vector, thereby doubling the vector dose applied to the animal (Figure S3A). We speculated that this would increase the number of *in vivo* transduced HSCs. To find the optimal timepoint for HDAd5/35++ injection, we measured hourly the number of HSCs in the peripheral blood of G-CSF/AMD3100-mobilized animals (between 4 and 12 h after AMD3100 injection). For each peripheral blood sample, we determined the percentage of CD34^+^/CD45RA^–^/CD90^+^ cells in PBMCs by flow cytometry (Figures S3B and S3D) and the percentage of progenitor colony-forming cells in CD34^+^ cells (as a functional HSC parameter) (Figure S3C). These tests showed a robust HSC mobilization with a peak at ∼8 h after AMD3100 injection. Based on this, we developed the mobilization/HDAd5/35++ injection regimen shown in Figure 1B.

In our *in vivo* HSC transduction studies, using the two-wave mobilization/HDAd injection protocol, mobilization efficacy was in the range of 18–70 × 10^3^ CD34^+^/CD45RA^–^/CD90^+^ cells per mL peripheral blood at the time of vector injection (Figure 1C).

#### Prophylaxis for cytokine responses

**Figure 2:**
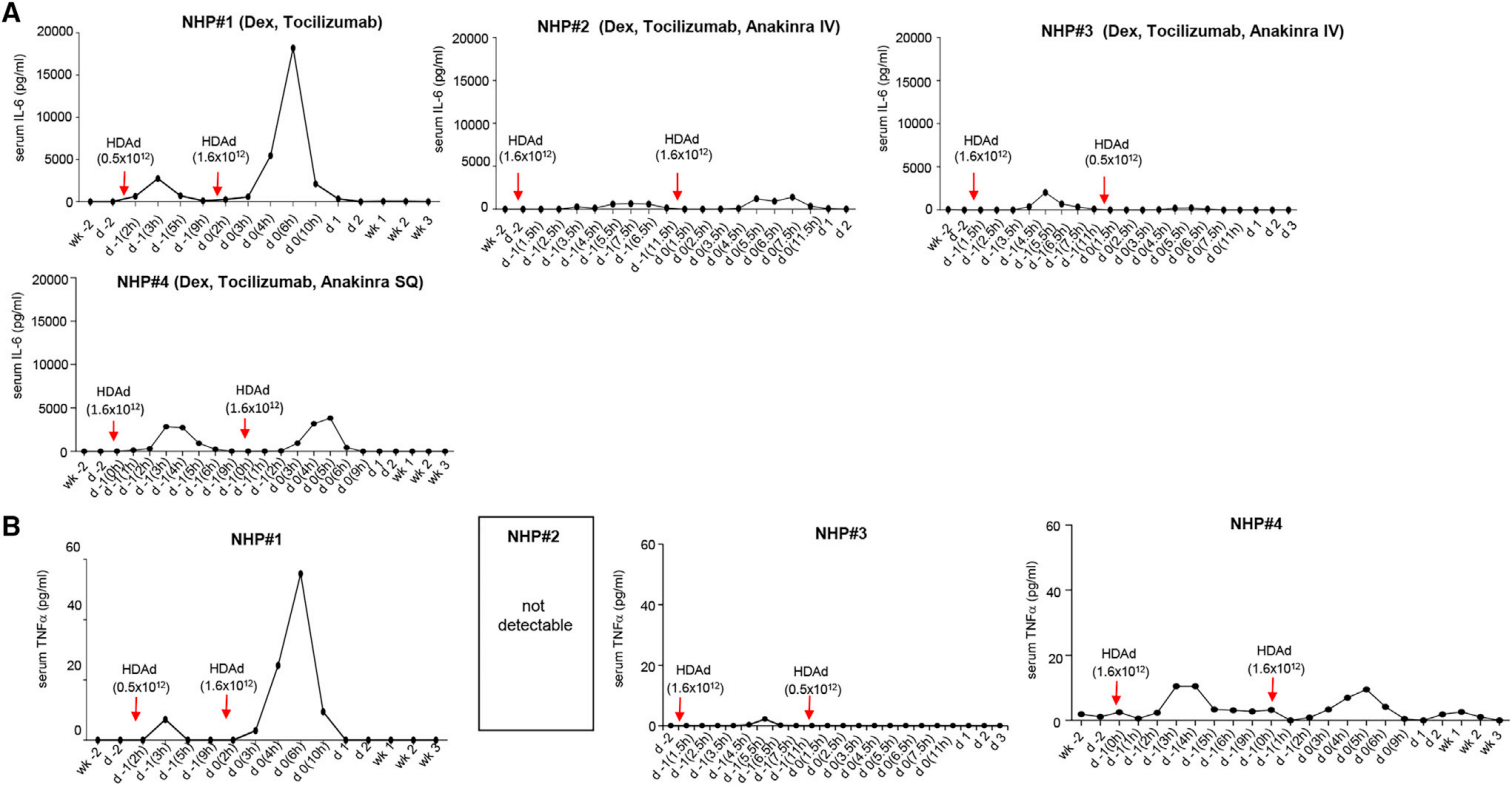
Serum levels of pro-inflammatory cytokines after HDAd5/35++ injection NHP#1 received dexamethasone (Dex) and tocilizumab. NHPs#2–#4 were pre-treated with dexamethasone, tocilizumab, and anakinra. In NHP#4, anakinra was given subcutaneously. The graphs also show the HDAd5/35++ vector dose injected. (A) IL-6 levels measured by cytometric bead array (CBA). (B) TNFα levels measured by CBA. TNFα was not detectable in NHP#2. IL-2, IL-4, IL-5, and IFNγ were not detectable by CBA in all animals.

A major risk factor with intravenous administration of adenovirus vectors is the activation of the innate immune system.^38^^,^^39^ A hallmark and causative factor of the innate immune response is the elevation of proinflammatory cytokines and chemokines, in particular IL-1 and IL-6.^40^, ^41^, ^42^ For example, intravenous injection of 1 × 10^13^ vp/kg of an HDAd5 vector into a nonmobilized baboon triggered serum IL-6 levels greater than 35,000 pg/mL and was accompanied by lethal acute toxicity.^39^ We previously showed that pre-treatment with dexamethasone blunted cytokines responses in mice.^12^ Here, we pre-treated NHP#1 with dexamethasone and the anti-IL6R monoclonal antibody tocilizumab (Figures 2A and 2B). This regimen was not sufficient to completely suppress the release of IL-6 and tumor necrosis factor (TNF)α, which peaked at 6 h after vector dosing. Further addition of the IL-1 blocker anakinra almost completely blunted cytokine responses associated with intravenous HDAd5/35++ vector administration at a total dose of 3.2 × 10^12^ vp/kg in NHPs#2 and #3. In NHP#4, the route of anakinra administration was changed from intravenous to subcutaneous, which is clinically more accepted. Furthermore, formulating HDAd in a buffer that did not contain glycerol and giving an intravenous (i.v.) saline bolus injection after infusion of HDAd5/35++ prevented hypotension and nausea (see materials and methods).

#### Immunosuppression

In contrast to *ex vivo* HSC gene therapy protocols that involve myeloablation/conditioning, in our *in vivo* approach, recipients of the therapy are fully immunocompetent. In our studies, we therefore had to suppress adaptive immune responses against nonrhesus transgene products expressed from the HDAd5/35++ vectors (human γ-globin, human mgmt^P140K^, SB100x, Flpe). In NHP#1, we used a combination of daily tacrolimus, sirolimus, and mycophenolate mofetil (MMF). However, this approach led to drug-related toxicities and weight loss in the animal, so NHPs#2–#4 received only tacrolimus or tacrolimus + MMF/abatacept (Table 2).

### Efficacy

#### HDAd5/35++ vector clearance from serum and PBMCs

The number of vector genomes per mL serum was measured by qPCR using mgmt^P140K^ or γ-globin-specific primers (Figure S4A). At 2 h after i.v. injection of 1.6 × 10^12^ vp/kg, vector genomes per mL serum were in the range of 6–17 × 10^6^ in NHPs#2, #3, and #4. By 10 h, the vast majority of vector genomes had been cleared from the serum. There was a long shoulder that had two pronounced peaks in NHP#4. Analysis of the vector copy number (VCN) in PBMCs early after HDAd5/35++ injection showed a peak with ∼4 vector copies per cell at ∼6 h (Figure S4B). Vector signals declined by day 7 to below 1 copy per cell, most likely due to the natural turnover of differentiated blood cells and loss of episomal vector DNA.

#### Preferential *in vivo* transduction of HSCs

**Figure 3:**
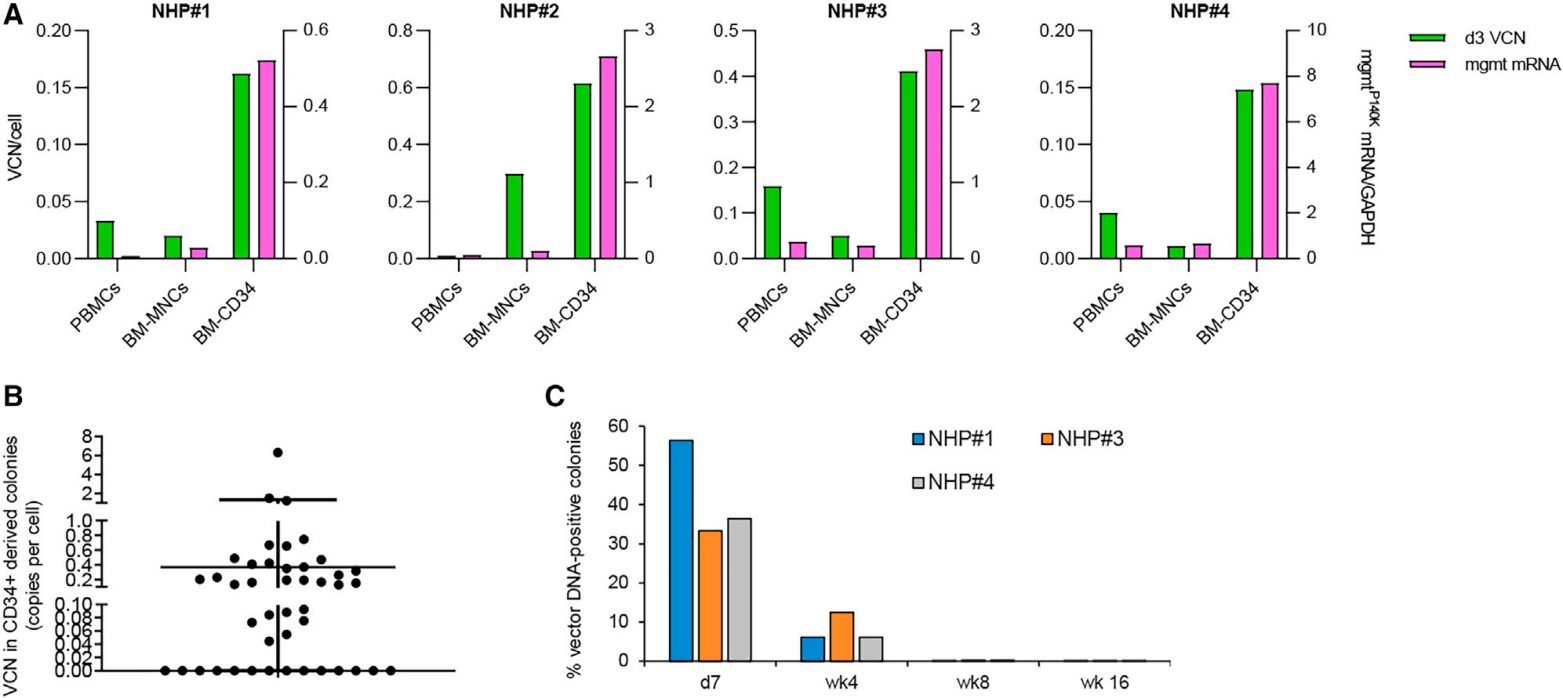
Vector genomes in HSCs (A) Vector copy number (VCN) (green) per cell at day 7 (NHPs#1, #2, and #4) and day 3 (NHP#2) after the second HDAd5/35++ injection. Genomic DNA was isolated from PBMCs, total bone marrow mononuclear cells (BM-MNCs), and BM CD34^+^ cells and subjected to qPCR. The mgmt^P140K^ mRNA levels in corresponding samples were measured by qRT-PCR (magenta). (B) Vector copies per cell (mean ± SD) in individual progenitor colonies. CD34^+^ cells from day 7 BM of NHP#3 were plated for progenitor colony assay. Colonies were picked after 12 days of culture and subjected to qPCR for vector genomes. (C) Percentages of vector-DNA-positive colonies in NHPs#1, #3, and #4 at the indicated timepoints.

A more detailed VCN analysis at day 7 (day 3 for NHP#2), including total bone marrow mononuclear cells (MNCs) and bone marrow CD34^+^ cells demonstrated preferential *in vivo* transduction of CD34^+^ HSCs (Figure 3A). The VCN in blood and bone marrow cells correlated with mRNA levels for mgmt^P140K^ relative to GAPDH mRNA (Figure 3A). To estimate the percentage of *in vivo*-transduced HSCs, we plated CD34^+^ cells (isolated on day 7 from the bone marrow of NHPs#1, #3, and #4) and measured the VCNs in individual progenitor colonies. Vector DNA was detectable in 55% of colonies (reflecting the *in vivo* transduction rate of HSCs) (Figure 3B). The vector copy in colonies ranged from 0.05 to 6 copies per cell, with the majority of colonies having around 0.5 copies. HDAd5/35++ genomes are episomal and are lost during cell division.^12^ This explains VCNs below 1.0 in the pool of cells from a given colony (Figure 3B). The VCN was analyzed in colonies at 4, 6, and 16 weeks after *in vivo* transduction (Figure 3C). Unexpectedly, the VCN declined over time.

#### γ-globin expression

γ-globin expression in peripheral RBCs was measured by (1) flow cytometry after intracellular staining with a (cross-reacting) anti-human γ-globin antibody in Nuclear Red-negative cells, (2) high-performance liquid chromatography (HPLC) of RBC lysates that can separate α2-, α1-, γ1-, and γ2-globin chains, and (3) qRT-PCR for human γ-globin mRNA (expressed as the percentage of γ-globin mRNA to rhesus α1-globin mRNA).

**Figure 4:**
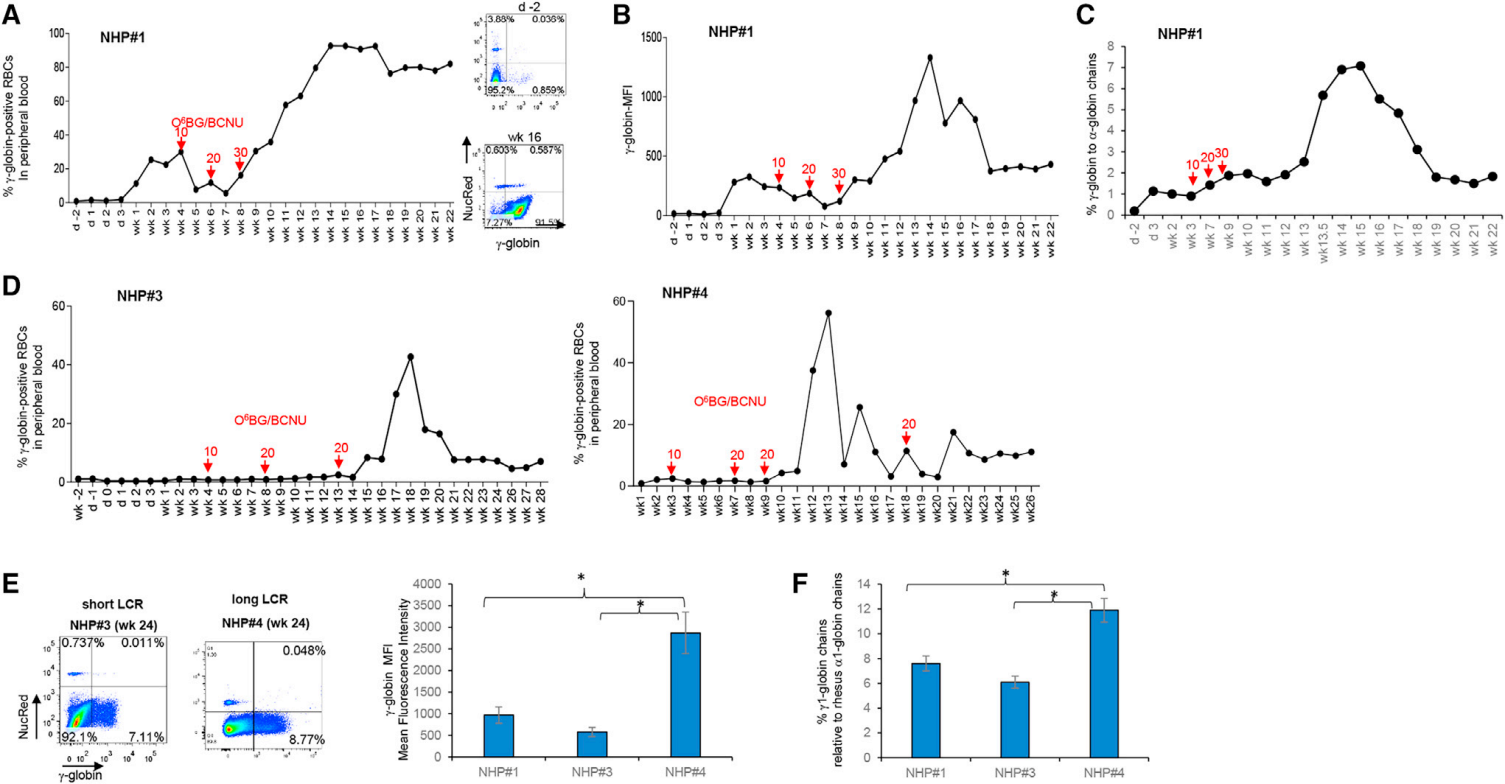
γ-globin expression in RBCs (A–C) NHP#1 data. (A) Percentages of γ-globin-positive RBCs in peripheral blood measured by flow cytometry. RBCs don’t contain nuclei and are negative for Nuclear Red (NucRed) staining. The time and dose of O^6^BG/BCNU selection are indicated. O^6^BG was given twice at 120 mg/m^2^. The BCNU doses were 10, 20, and 30 mg/m^2^. (B) γ-globin mean fluorescence intensity (MFI) in peripheral blood samples. (C) Percentage of γ-globin chains relative to rhesus α1-globin measured by HPLC. (D) γ-globin expression in RBCs of NHPs#3 and #4. Note that the aggressive immunosuppressive regimen (tacrolimus, sirolimus, and MMF) and *in vivo* selection (10, 20, and 30 mg/m^2^ BCNU, 2 weeks apart) performed in NHP#1 caused critical body weight loss and was therefore not repeated in the subsequent animals. Shown are percentages of γ-globin-positive RBCs in peripheral blood measured by flow cytometry. (E and F) Level of γ-globin expression from “short” LCR (NHPs#1 and #3) and “long” LCR (NHP#4). (E) MFI data. Left panel: representative γ-globin/NucRed flow plots. Right panel: summary of γ-globin MFIs (mean ± SD). ∗p < 0.05. (F) HPLC: γ-globin to rhesus α1-globin chains are shown for NHP#1 (week 19), NHP#3 (week 18), and NHP#4 (week 15) (mean ± SD). ∗p < 0.05.

In NHP#1, γ-globin became detectable by flow cytometry of peripheral RBCs after week 2 (Figure 4A). After the third cycle of O^6^BG/BCNU treatment, the percentage of γ-globin^+^ RBCs climbed to 90%, remained stable for 1 month, and then declined to ∼75% by week 23 (the endpoint of the study). The γ-globin mean fluorescence intensity (MFI) followed the pattern of γ-globin marking with a more pronounced decline observed after the peak at week 16 (Figure 4B). HPLC data agreed with this kinetic (Figures 4C and S5). At the peak, γ-globin was 8% of rhesus α1-globin chains.

Notably, NHPs#3 and #4 received a less intense *in vivo* selection and immunosuppression regimen (see Table 2). Overall, the kinetics of γ-globin-marking in peripheral RBCs was similar in these animals, with a sharp increase after the completion of the third O^6^BG/BCNU selection cycle, with peak levels of 48% and 57% γ-globin^+^ RBCs for NHPs#3 and #4, respectively (Figure 4D). γ-globin-marking then declined and reached stable levels at ∼10%.

The HDAd5/35++ vector injected into NHP#4 contained the long β-globin LCR, which conferred higher γ-globin expression levels, as reflected by a 3- to 4-fold higher MFI (Figure 4E) and significantly higher percentages of human γ-to rhesus α1-globin chains measured by HPLC (Figure 4F).

The levels and kinetics of γ-globin mRNA in RBCs roughly followed the pattern of γ-globin protein chains with an increase by *in vivo* selection (Figure S6). Interestingly, in NHPs#3 and #4, γ-globin mRNA levels did not decline, as seen on the protein level.

**Figure 5:**
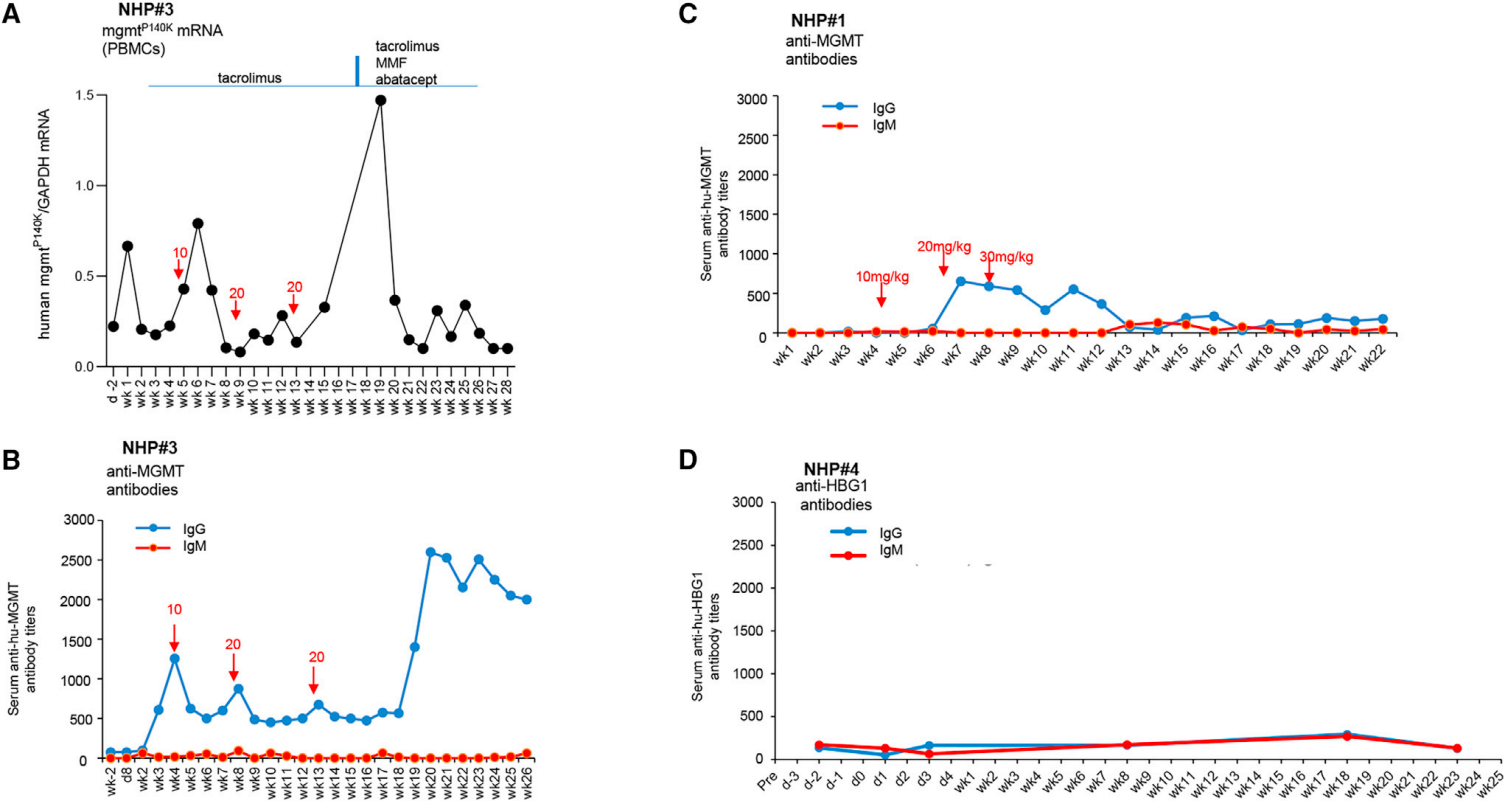
Serum IgG and IgM antibody titers (A and B) Correlation of mgmt^P140K^ mRNA expression in PBMCs, immunosuppression (A), and anti-human MGMT serum antibodies (B) in NHP#3. Note that mgmt^P140K^ mRNA levels increased after the third round of *in vivo* selection and the implementation of a more stringent immunosuppressive regimen (tacrolimus, MMF, and abatacept). Increase in MGMT expression coincided with an elevation of serum antibodies, i.e., a stronger immune response, which in turn could have eliminated transduced PBMCs causing the decline in human mgmt^P140K^ mRNA levels. (C) Anti-human MGMT titers in NHP#1, the animal that received more stringent immunosuppression, in comparison to NHP#3. (D) Anti-human HBG1 titers in NHP#4 as a representative example.

We also measured mRNA levels of the other transgene, human mgmt^P140K^, which is under the control of the EF1α promoter (Figures 5A and S7). In PBMCs, *in vivo* selection increased the level of mgmt^P140K^ mRNA in NHPs#1, #3, and #4. As seen with γ-globin, the increase in mgmt^P140K^ mRNA levels was followed by a 5- to 10-fold decline. mgmt^P140K^ mRNA in bone marrow (BM) MNCs and CD34^+^ cells did not increase by *in vivo* selection.

We hypothesized that a critical factor that played a role in the decline of globin marking after the peak was immune responses against cells that express transgene products, specifically human MGMT^P140K^. As shown for NHP#3, the decline in γ-globin^+^ RBCs coincided with a rise in serum anti-human MGMT immunoglobulin G (IgG) antibodies (Figures 5A and 5B). The decline could temporarily be reversed with more intense immunosuppression including MMF and abatacept, indicating that an immune response was responsible for the decline. Note that anti-MGMT titers are lower in NHP#1, which received a more stringent immunosuppression (Figure 5C). Human and rhesus HBG1 proteins are 98% identical, and no profound anti-human HBG1 humoral immune response was detected (Figure 5D).

In summary, the γ-globin expression analysis in RBCs shows that it is possible to achieve meaningful marking rates with *in vivo* selection and immunosuppression. As shown in NHP#1, it can reach 90% γ-globin^+^ RBCs and γ-globin levels greater 10% of rhesus α1-globin levels. In contrast to peripheral RBCs and PBMCs, in BM-MNCs and CD34^+^ cells, the VCN and level of mgmt^P140K^ mRNA were low and declining over time. This implies that peripheral γ-globin^+^ RBCs are derived from HSCs that do not reside in the BM.

#### The spleen as a homing site for *in vivo*-transduced HSCs

**Figure 6:**
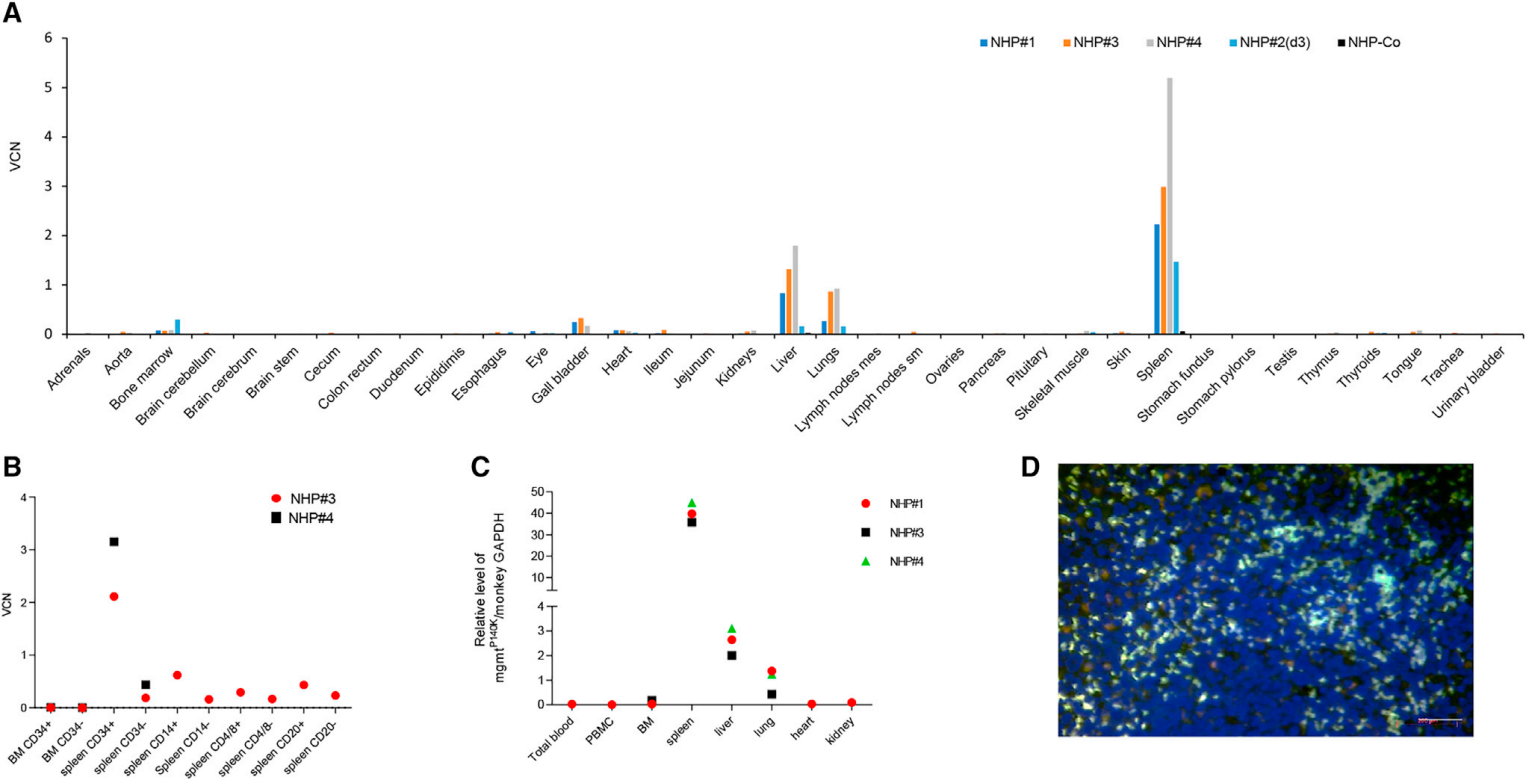
Vector biodistribution in other tissues (A) VCN. NHPs#1, #3, and #4 were euthanized at ∼6 months after HDAd5/35++ injection, and tissues were analyzed. NHP#2 was euthanized at day 3 after HDAd5/35++ injection. NHP-Co (black bars) was a rhesus macaque that was not mobilized but injected with HDAd5/35++ (twice 1.6 × 10^12^ vp/kg) and euthanized at day 3. (B) VCN in BM and spleen subfractions of NHPs#3 and #4. (C) Relative level of mgmt^P140K^ mRNA in tissues. (D) Spleen section from NHP#3 stained with antibodies against human MGMT (green). The scale bar is 20 μm. Controls are shown in Figure S9.

During necropsy of NHPs#3 and #4 performed ∼6 months after HDAd5/35++ injection, blood was washed out from the circulation, and tissues were collected. The VCN per cell was measured by qPCR. The highest VCNs (2–5 copies per cell) were found in the spleen, followed by the liver, lung, and gall bladder (Figure 6A). Analysis of a control animal that was not mobilized and only i.v. injected with HDAd5/35++ (twice 1.6 × 10^12^ vp/cell) (Figure 6A; “NHP-Co”) presented detectable vector DNA only in the spleen (0.06 copies/cell) and liver (0.03 copies/cell), levels that were 100-fold lower than in NHPs#3 and #4. This indicates that transduced cells detected in the spleen are derived from mobilized HSCs that returned to the spleen and not from direct virus transduction. Furthermore, VCNs from NHP#2 (euthanized at day 3) show homing to spleen (VCN: 1.47) and BM (VCN: 0.3). VCNs in spleen were 4- to 7-fold higher for NHPs#3 and #4, which underwent *in vivo* selection, indicating that transduced cells expanded over time in the spleen as a result of *in vivo* selection. Notably, scant to mild mixed extramedullary hematopoiesis was seen on spleen samples collected at necropsy (see pathology report in supplemental information).

Pools of splenocytes from NHPs#3 and #4 were sub-fractioned by magnetic cell isolation using CD34, CD14, CD4/CD8, and CD20 antibody beads (Figure 6B). The highest VCN was found in spleen CD34^+^ cells (2 and 3.2 copies/cell in NHPs#3 and #4, respectively) compared with 0.19 and 0.44 vector copies in splenic CD34-negative cells, including CD14^+^, CD20^+^, and CD4^+^/8^+^ cells. In contrast, the VCN in BM was barely above the detection limit. A similar distribution in tissues was seen based on the mgmt^P140K^ mRNA (Figure 6C). Transduced splenocytes were also detected on tissue sections by immunofluorescence for human MGMT, with about 25% of MGMT-positive cells on a given spleen section being positive (Figures 6D and S8A). Sparse MGMT-positive cells were found in liver and lung sections, which did not resemble hepatocytes or alveolar epithelial cells (Figure S8B). Importantly, no vector genomes or mgmt^P140K^ mRNA were found in reproductive organs in all four animals.

#### Contribution of splenic HSCs to hematopoiesis

In agreement with published literature,^43^ the number of CD34^+^ cells per cm^3^ tissue was 10-fold lower for the spleen than for the BM (Figure S9A). CD34^+^ cells isolated from rhesus spleen were functional, as shown by their capacity to expand and differentiate *in vitro*. Their ability to form progenitor colonies was comparable to that of the CD34^+^ cells purified from BM (Figure S9B). When subjected to erythroid differentiation (ED) in liquid culture,^44^^,^^45^ erythroblasts developed, and at a later stage (day 10 of ED), efficient rhesus hemoglobin (HBA and HBF) synthesis and enucleation were observed (Figures S9C and S9D). Importantly, the percentage of γ-globin^+^-enucleated cells and the γ-globin MFI were greater in cells differentiated from CD34^+^ cells of NHP#4 compared with those from an untreated animal. This could be due to added human γ-globin production in erythroblasts derived from gene-modified CD34^+^ cells. The latter is supported by the presence of human HBG mRNA in NHP#4 samples (Figure S9D). Together, these data indicate that *in vivo*-transduced splenic HSCs have the potential to contribute to hematopoiesis and be the source of γ-globin^+^ RBCs in peripheral blood.

### Safety related to HDAd5/35++ injection into mobilized rhesus macaques

#### Pathology/histopathology

**Figure 7:**
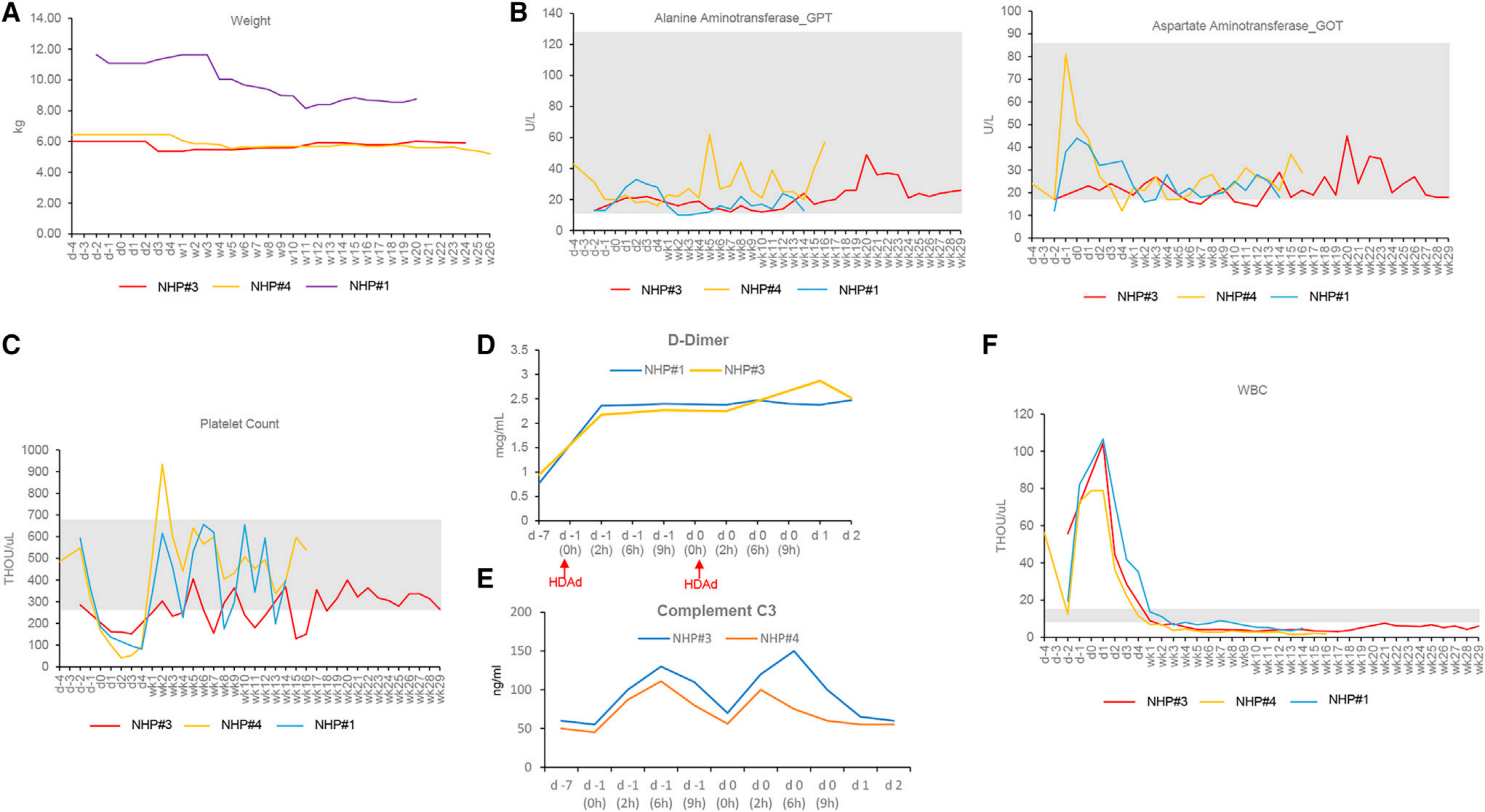
Weight and selected hematological parameters WBCs, white blood cells.

During the mobilization and HDAd5/35++ injection procedures, all animals were BAR (bright, alert, reactive) without an elevation of body temperature or a loss of appetite. While we observed significant body weight loss in NHP#1 due to an intensive immunosuppressive regimen with tacrolimus, sirolimus, and MMF, in addition to the *in vivo* selection (BCNU dose: 30 mg/m^2^) (Figure 7A), the overall physical condition of the other animals was good, and no remarkable, treatment-related clinical side effects were listed in the audited pathology/histopathology reports (see supplemental information).

#### Hematology

Most hematological and biochemical parameters (complete blood count [CBC] and blood chemistry) were within normal range, including the liver transaminases GPT and GOT (Figures 7B and S10). Noteworthy are the following abnormalities: (1) Temporary thrombocytopenia was observed between days 1 and 4, which was followed by a compensatory increase in platelet counts (Figure 7C). (2) Elevation in D-dimer concentrations to levels ∼2.5 μg/mL (but below the critical 5 μg/mL value) was observed starting immediately after HDAd5/35++ injection and lasting for 3 days (Figure 7D). Other coagulation parameters (fibrinogen, prothrombin time, partial thromboplastin time) were unremarkable (Figure S10, part 5). (3) The concentration of complement factor C3 increased, reaching peak values (100–150 ng/mL) at 6 h after HDAd5/35++ injection (Figure 7E). (4) As expected, G-CSF/AMD3100 mobilization resulted in an increase in white blood cell counts (lymphocytes, neutrophils, monocytes, basophils), from days 0 to 4 (Figures 7F and S10, part 3). Notably, neutrophils contain pro-inflammatory cytokines that can be released during senescence. To counteract this, we gave anakinra also on days 1 and 2 after HDAd5/35++ injection (see materials and methods). (5) Lymphocyte counts were low due to immunosuppression (Figure S10, part 3).

We also measured by flow cytometry the percentage of lineage-positive (CD3^+^, CD14^+^, and CD20^+^) cells in PBMCs and BM-MNCs (Figure S11). In PBMCs, a transient decline in the percentage of CD3^+^ and CD20^+^ cells from day −2 to weeks 3–4 and a slight increase in CD14^+^ cells were noticeable in all four animals. The number of BM CD34^+^ cells transiently declined after O^6^BG/BCNU treatment as a result of *in vivo* selection.

#### Adaptive immune responses

In spite of immune-suppression, humoral immune responses (serum IgG and IgM) were activated. Anti-human MGMT and anti-human γ-globin serum antibody titers for selected animals were shown in Figure 5. The full set of data can be found in Figures S12 and S13. Antibody responses against HDAd5/35++ virions (Figure S14), SB100x, and Flpe (Figure S15) were transient, most likely due to the short-term presence of the corresponding antigens. In future studies, it needs to be tested whether immunosuppression (e.g., by tacrolimus + MMF) is needed to prevent adaptive immune responses against the transiently expressed SB100x transposase (fish) and Flpe recombinase (*E. coli*). Clearly, to avoid immune responses in our upcoming NHP studies, HDAd5/35++ vectors should contain only rhesus transgenes.^46^

#### *In vivo* selection

In our studies, *in vivo* selection with O^6^BG/BCNU resulted in an increase of transgene-marked cells with acceptable hematopoietic and extramedullary toxicity. These safety data are in agreement with previous studies in mice, dogs, NHPs, and humans involving the transplantation of *ex vivo* lentivirus-vector-transduced HSCs and low doses of methylating agents such as O^6^BG/BCNU and the clinically approved drug temozolomide.^23^^,^^24^^,^^47^^,^^48^

#### SB100x-mediated integration

SB100x and Flpe are expressed from an episomal vector (HDAd5/35++-SB) that will be lost during cell division. While Flpe and SB100x mRNA was detectable at day 3 in CD34^+^ cells, levels of these mRNAs sharply declined by day 8 and were undetectable by week 4 post-transduction (Figure S16A). The kinetics of mRNA expression in PBMCs was similar, with a peak around day 1 and a loss by week 4 (Figure S16B).

**Figure 8:**
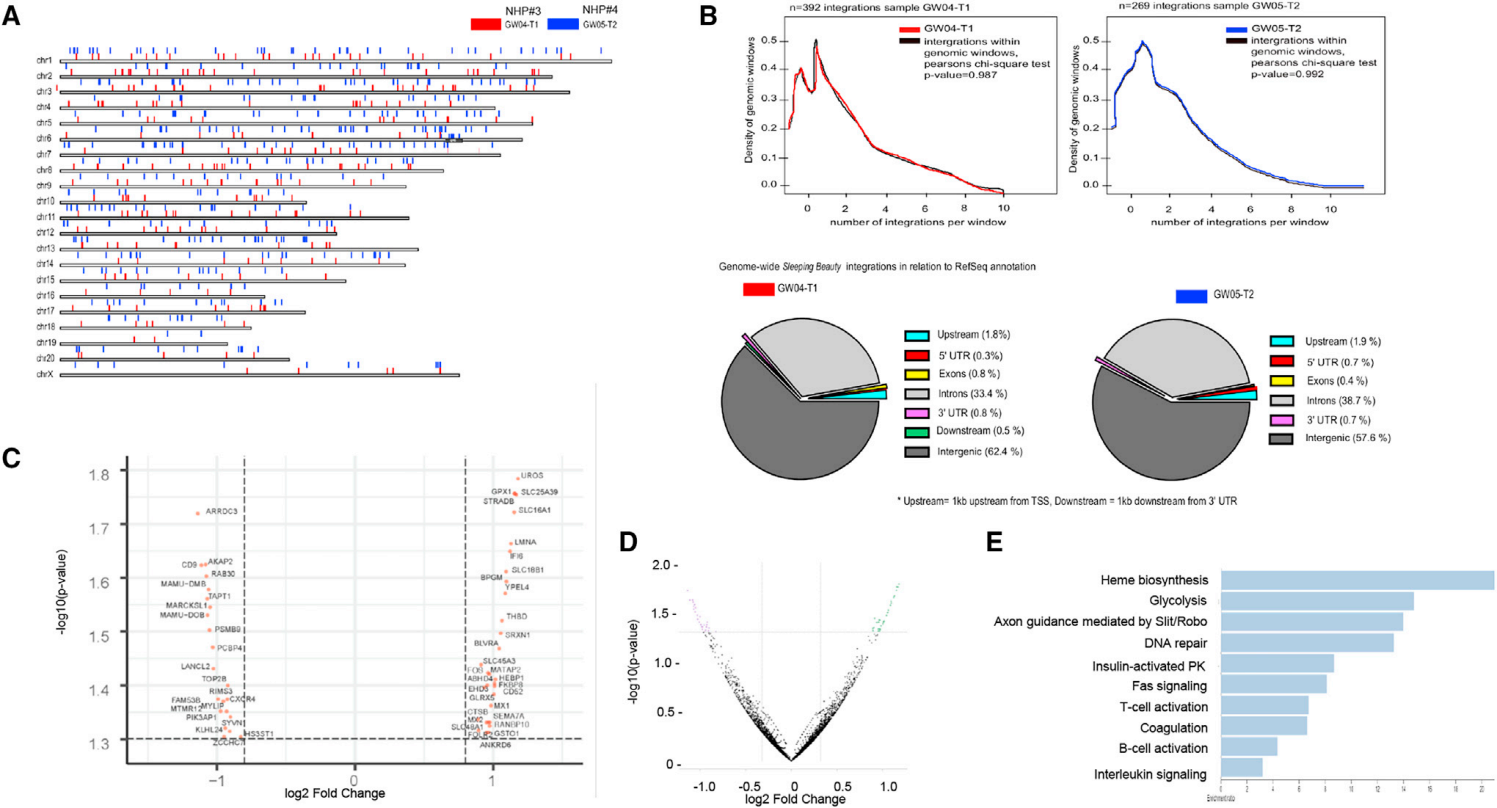
Genome and transcriptome analysis in NHPs#3 and #4 (A) Chromosomal distribution of integration sites in splenic CD34^+^ cells harvested at ∼6 months. The integration sites are marked by vertical red lines. GW04-T1 = NHP#3 and GW05-T2 = NHP#4. (B) Upper panel: integration pattern in rhesus genomic windows. The number of integrations overlapping with continuous genomic windows and randomized mouse genomic windows and size were compared. This shows that the pattern of integration is similar in continuous and random windows. Maximum number of integrations in any given window was not more than 3, with one integration per window having the higher incidence. Lower panel: integration sites were mapped to the genome, and their location with respect to genes was analyzed. Shown is the percentage of integration events that occurred 1 kb upstream of transcription start sites, 3′ UTR of exons, protein coding sequences, introns, 3′ UTRs, 1 kb downstream of 3′ UTR, and intergenic. (C) mRNA from total blood cells of NHP#3 was subjected to RNA-seq performed by Omega Bioservices. Shown are genes with altered mRNA expression (log2 fold change) ranked based on their p value. (D) Volcano plot of all mRNA data. (E) Panther pathway overrepresentation analysis.

The distribution of integration sites over the rhesus genome in splenocytes of NHPs#3 and #4 is shown in Figures 8A and 8B. The vast majority of integrations were within intergenic (62.4/57.6%) and intronic regions (33.4/38.7%) for NHPs#3 and #4, respectively. The integration was random, without preferential integration in any given window of the whole rhesus genome. No integration within or near any proto-oncogene was found. No accumulation of integration sites indicating clonal expansion of transduced splenocytes was found. This SB100x-mediated integration pattern is in agreement with previous studies.^12^^,^^16^^,^^49^, ^50^, ^51^, ^52^ Notably, the VCN in BM-MNCs at necropsy was too low for an integration site analysis.

Furthermore, we performed genome-wide RNA sequencing (RNA-seq) on spleen RNA from NHP#4 to assess the effects on the transcriptome. We found a modestly altered expression of only 53 genes (Figures 8C–8E). None of these genes was proto-oncogenic or related to cancer pathways. This indicates that SB100x and O^6^BG/BCNU treatments do not exert changes in gene expression in a 6-month study.

## Discussion

Here, we show that our new *in vivo* HSC transduction/selection approach is safe in rhesus macaques and yields human γ-globin^+^ RBCs for 6 months, i.e., the length of the study. Long-term γ-globin marking levels (80%, 7%, and 11% for NHPs#1, #3, and #4, respectively), which were reached in our study, would be therapeutic in patients with hemoglobinopathies.

Our studies suggest that CD46 is a suitable receptor to target primitive HSCs. Other than CD46 transgenic mice, NHPs appear to be the only other adequate large animal model for pre-clinical studies with CD46-interacting HDAd5/35++ vectors**.** Canine^53^ and pig CD46 are not recognized by these vectors**.** A major limitation of NHPs is, however, that unlike humans, rhesus erythrocytes possess CD46 on their surface^30^ and therefore cause unspecific sequestration/loss of i.v.-injected HDAd5/35++ vector particles. On the other hand, recent data by Hemminki et al. indicate that adenovirus-binding to erythrocytes is reversible.^54^ The slow (biphasic) serum clearance of vector genomes (Figure S4), together with our previous observation that the binding of ligands, including HAd5/35++, to CD46 results in the shedding of the extracellular domain of CD46,^55^ could indicate that this observation is correct. However, it is unknown whether HDAd5/35++ virions dissociated from RBCs are still infectious. To saturate CD46 on erythrocytes, we injected high vector doses in two cycles on consecutive days. (Notably, HDAd5/35++ vectors can be easily produced at high yields and low costs.)

A major focus of this study was to assess the safety of our approach. As is known, i.v. injection of serotype 5 adenovirus vectors is associated with transaminitis.^38^^,^^39^ We designed our HDAd5/35++ vector to avoid transduction by using short fiber shafts that block hepatocyte transduction. So, liver function parameters in our NHP studies remained within the normal range. To minimize any side effects from cytokine release, animals received a short course of a combination of dexamethasone, tocilizumab, and anakinra, all FDA-approved drugs that are currently being used in the clinic to prevent or treat cytokine release in chimeric antigen receptor T cell therapies. With this triple drug prophylaxis regimen, the infusion of high HDAd5/35++ doses (total 3.2 × 10^12^ vp/kg) was well-tolerated, and there were no clinical side effects. Notable deviations from normal hematological parameters were (G-CSF-triggered) leukocytosis, mild transient complement activation, and an increase in D-dimers without any symptoms. In addition, there was a brief (2–4 days) decrease in platelets, which was likely multifactorial.

Because of recent reports of rare myeloid malignancies associated with lentivirus vector integration in clinical trials,^56^ we paid specific attention to potential genotoxic effects in the context of SB100x-transposase integration and the mgmt^P140K^-based selection systems. In the genome-wide integration site and transcriptome studies, we confirmed random integration without clonal dominance and minimal changes in the mRNA profile due to integration into exons. This integration pattern is, theoretically, safer than that of lentivirus vectors, which show a preference for actively transcribed genes. Clearly, the risk of genotoxicity cannot be ignored and has to be studied more carefully based on longitudinal integration site analyses.

If *in vivo* selection (O^6^BG + 10→20→30mg/m^2^ BCNU) and an immunosuppression (sirolimus + tacrolimus + MMF) regimen were implemented in NHP#1, γ-globin-marking of peripheral RBCs reached 90% after the third round of *in vivo* selection. Based on RBC HPLC and mRNA data, the level of γ-globin chains was 8% of rhesus α1-globin chains. However, the combination of *in vivo* selection with this intensive immunosuppression regimen resulted in body weight loss. Thus, subsequent animals (NHPs#3 and #4) received only tacrolimus and MMF, and we also reduced the third dose of BCNU to 20 mg/m^2^. With this regiment, γ-globin marking peaked at ∼50% after *in vivo* selection, with a subsequent decline that leveled out at a stable marking rate of ∼10% γ-globin^+^ RBCs. This decline was most likely due to an immune response against human transgene products, specifically human MGMT^P140K^. Immune responses could result in a loss of transduced (marked) cells or also a downregulation of expression at the levels of translation, as recently shown in recombinant adeno-associated virus (rAAV) gene therapy studies.^57^ The latter could explain the discrepancy between declining RBC marking and increasing γ-globin mRNA levels in RBCs, specifically in NHPs#3 and #4. γ-globin levels in RBCs were higher with a “long” ∼26 kb β-globin LCR driving γ-globin expression compared to a 4.3 kb LCR version. Notably, the “long” LCR has the capacity to reduce the position effects of integration.^13^

Measurement of vector genome copies confirmed that HDAd5/35++ vectors preferentially transduced mobilized HSCs in the periphery. At day 7, after vector injection, 30%–55% of colony-forming CD34^+^ cells in the BM were positive for vector DNA by qPCR. However, in all animals, the VCN in BM-MNCs and CD34^+^ cells declined regardless of *in vivo* selection. A similar tendency was seen for BM mgmt^P140K^ mRNA levels. This observation was in conflict with γ-globin-marking in RBCs and mgmt^P140K^ mRNA expression in PBMCs. Consequently, BM HSCs cannot be the major source for genetically modified cells in the periphery. Vector DNA and RNA analyses in other tissues pointed toward a contribution of splenic HSCs to hematopoiesis and transgene-expressing peripheral blood cells. In the spleen, we found a VCN of 5 copies per cell and a high level mgmt^P140K^ mRNA expression that was also reflected by 25% MGMT-positive splenocytes found by immunofluorescence analyses. We postulate that the high VCN/mgmt^P140K^ expression in the spleen was the result of (1) mobilized, transduced HSCs efficiently returning and surviving in the spleen and (2) an expansion by O^6^BG/BCNU selection. Data supporting the first claim come from the finding that i.v. HDAd5/35++ injection into a nonmobilized rhesus macaque resulted in only a minimal transduction of the spleen (Figure 6A; “NHP-Co”), which is in agreement with previously published biodistribution data in NHPs after injection of Ad5/35++^31^ and Ad35 vectors.^32^ The second claim is based on the finding that the VCN in the spleen in NHP#2 (day 3, no *in vivo* selection) is lower than in NHPs#1, #3, and #4 (6 months, with *in vivo* selection). Furthermore, we hypothesize that transduced splenic HSCs contribute to peripheral blood cells, which is more evident in gene-modified RBCs compared with PBMCs (because RBC numbers in peripheral blood are 1,000-fold higher than PBMC numbers)*.* To support our hypothesis, we subjected CD34^+^ cells, isolated from NHP#4 at 6 months, to *in vitro* ED. We showed a higher percentage of γ-globin^+^ erythroid cells and a higher MFI of γ-globin per cell than in control settings with CD34^+^ cells from a nontransduced rhesus. The number of splenic CD34^+^ cells isolated from a 1 cm^3^ spleen was at least 10-fold lower than that isolated from a similar volume of a BM-MNC pellets. Therefore, the contribution of splenic HSCs to differentiated blood cells (including γ-globin^+^ RBCs) should be correspondingly less.

Transduced cells (based on VCNs and immunofluorescence) were also found in the liver/gallbladder and the lung. Because of technical difficulties to isolate these cells, it is not clear whether they have HSC function. Notably, there were no remarkable pathological/histopathological alterations in these organs.

In contrast to mice, where we observed comparably high VCNs (∼3.5 copies per cell) in the spleen, BM, and PBMCs (see Figure S17), in NHPs we found a decline of VCN and mgmt^P140K^ mRNA in *in vivo*-transduced HSCs that returned to the BM. We believe that the most likely explanation for this finding is the immune-mediated destruction of transduced HSCs in the BM but not in the spleen. Notably, the spleen is thought to be immune-privileged due to the presence of large numbers of immunosuppressive and immune-tolerizing cells.^58^

Publications of HSC trafficking and hematopoiesis after mobilization with different agents are sparce.^43^^,^^59^ The homing of transplanted HSC is relatively well-studied in myeloablated mice and NHPs.^60^^,^^61^ It is thought that i.v. injected HSCs are not selectively attracted to the BM and spleen but initially are randomly distributed in tissues.^62^ Only in the BM and spleen do HSCs survive longer than 48 h because of the specific microenvironment constituted by β_1_ and α_4_ integrins and chemokines, specifically the CXCR4/SDF-1 pathway.^63^ The ability to mark mobilized HSCs *in vivo* by HDAd5/35++ vector transduction could help in studying their fate and improving BM homing*.*

While our approach has the potential to greatly simplify HSC gene therapy in humans, it currently has shortcomings. The first is related to the i.v. injection of viral particles, which results in vector sequestration by blood components and by the reticuloendothelial system of the liver and spleen. This, in turn, triggers innate toxicity and limits target cell transduction. Second, the level of stable HSC transduction is low, in part because it necessitates the co-infection of two vectors, the transposon vector and transposase vector. Reaching therapeutic marking rates therefore requires *in vivo* HSC selection with low-dose methylating drugs.

Our current efforts to address these shortcomings and further test our approach in NHPs include: (1) HSC mobilization using a 1-day regimen with truncated GRO-β and AMD3100.^7^ This will reduce leukocytosis and cytokine release from mobilized neutrophiles. A mobilization regiment without G-CSF would also be more appropriate for patients with sickle cell disease. (2) Targeting HDAd vectors to another receptor that is present on primitive HSCs to increase transduction and, in the case of NHPs, prevent RBC sequestration. This includes new HDAd vectors or fibers from alternative adenovirus serotypes. Serotype switching would also address the issues of pre-existing anti-Ad5 antibodies in humans and innate toxicity triggered by Ad5 penton or hexon proteins.^64^, ^65^, ^66^ (3) Directing more transduced HSCs to the BM than to the spleen because the BM is the major contributor to blood cells under physiological conditions. Potential ways to achieve this include short-term overexpression of cxcr4 on mobilized HSCs^67^ or treatment with nicotinamide.^68^ (4) Focus on genome-editor-based approaches (Base or Prime Editors) that would capitalize on an intrinsic, disease-background-related mechanism for the *in vivo* expansion of edited HSCs and progenitors without vector integration would require only one HDAd vector (unpublished data).

In summary, we showed clear evidence of successful *in vivo* HSC transduction; based on VCNs, γ-globin mRNA, mgmt^P140K^ mRNA, and γ-globin protein levels, as well as a response of these parameter to O^6^BG/BCNU *in vivo* selection. This, together with the good safety profile, suggests that *in vivo* HSC gene therapy could be feasible in humans upon further improvements, some of which are outlined above. We believe that our approach will be more efficient in humans than in rhesus macaques because (1) vector sequestration by RBCs will not have a critical impact on *in vivo* HSC transduction and BM homing, (2) immune responses against human transgene products will be absent, and (3) higher γ-globin expression levels are expected in patients where the pathologic background will generate a selective advantage for the transduced cells.

## Materials and methods

### HDAd5/35++ vectors

HDAd-SB, HDAd-mgmt/GFP, HDAd-γ-globin-hu-mgmt^P140K^, and HDAd-long-LCR-γ-globin-hu-mgmt^P140K^ were described earlier.^14^^,^^16^^,^^36^

### Animals

Three male and two female rhesus macaques (*Macaca mulatta*) from the Oregon National Primate Research Center were used for the studies (see Table 1). The studies were performed by the WaNPRC Research Support Team. All experiments were conducted in accordance with the institutional guidelines set forth by the University of Washington. The studies were approved by the University of Washington IACUC (protocol no. 3108-04). After implantation of an i.v. catheter, animals were housed individually.

### Antibiotics

CEFTAZIDIME/TAZICET (Hospira, Inc) was injected i.v. at 150 mg/kg on the day of surgery and continued through study as long as tether is in, semel in die (SID; once a day). FLUCONAZOLE (Northstar Rx) was administered per os (PO; orally) at 50 mg flat dose per animal starting on day –5, SID; ACYCLOVIR (AuroMedics Pharma) was delivered i.v. at 10 mg/kg starting on day –5, SID.

### Mobilization

FILGRASTIM/G-CSF/NEUPOGEN (Amgen) was injected subcutaneously (s.c.) at 50 mcg/kg in p.m. from day –5 to day 0 (SID). AMD3100/Plerixafor (Calbiochem) was given s.c. at 5 mg/kg at midnight on days –1 and 0 (8 h before HDAd5/35++ dosing).

### Cytokine prophylaxis

DEXAMETHASONE (Fresenius Kabi, USA) was injected i.v. (4 mg/kg), at 2 p.m. on day –2 and then two doses each on days −1 and 0. ANAKINRA/KINERET (Swedish Orphan Biobivitrium) was administered s.c. at 50 mg/dose flat dose, 2 doses each on days –1 and 0 (1 h before and 6 h after HDAd5/35++ injection), one dose each on days 1 and 2. TOCILIZUMAB/ACTEMRA (Genentech, Inc) was injected i.v. at 8 mg/kg, 2 doses each on days –1 and 0 (1 h before and 6 h after HDAd injection) via diluted infusions (50 mL each).

### HDAd5/35++ vector infusion

For infusion, HDAd preps were thawed, diluted with PBS (room temperature), and infused within 30–60 min after preparation. A low dose (5 × 10^10^ vp/kg in 5 mL of PBS was given over 10 min followed by the therapeutic dose (usually 1.6 × 10^12^ vp/kg) in 20 mL of PBS infused over 20 min.

### Anti-emetic/hypotension prophylaxis

Lactated Ringer Solution/LRS BOLUS was injected i.v. (8 mL/kg) on days –1 and 0 (SID), give after second HDAd5/35++ infusion over 15 min. MAROPITANT (CERENIA) was given i.v. at 1 mg/kg on days –1 and 0 (SID) prior to HDAd5/35++ infusions. ONDANSETRON (Hikma Pharmaceutricals) was delivered i.v. at 2 mg/kg on days –1 and 0 (SID) prior to HDAd5/35++ infusions.

### Immunosuppression

TACROLIMUS/PRPGRAF (Astellas Pharma) was administered at a starting dose of 0.02 mg/kg on day –5 morning, and then adjusted to get blood level ∼15 ng/mLand continued through study, bis in die (BID; twice a day). Mycophenolate Mofetil Hydrocloride/MMF/CellCept (Genentech) was given via PO (20 mg/kg; BID) on day 1. ORENCIA/ABATACEPT (Brystol-Myers Squibb) was given i.v. at 60 mg/dose flat dose on days –2, 0, 2, 7, and 14.

### *In vivo* selection

O^6^-benzylguanine (Sigma) and carmustine/BCNU (Sigma) were made fresh for each injection. First, O^6^BG (120 mg/m^2^) was infused i.v. over 15 to 20 min (flow rate ∼600 mL/h). BCNU was given ∼30–45 min after the end of O^6^BG infusion. The BCNU doses were 10, 20, and 30 mg/m^2^ as indicated. O^6^BG infusion (120 mg/m^2^) was repeated 7–8 h after the end of the first infusion. Neutrophil counts decreased after each round of O^6^BG/BCNU treatment. The subsequent dose of O^6^BG/BCNU was therefore given only after neutrophil counts recovered (2–4 weeks).

### Necropsy

Animals were sedated and then injected i.v. with an overdose of pentobarbital. Blood was flushed out from the body with 5 L of PBS using an external perfusion pump.

### Further details

The following methods can be found in the supplemental information: HDAd5/35++ production, CD34^+^ cell culture; isolation of CD34^+^ cells from the spleen and BM; *in vitro* ED of rhesus CD34^+^ cells, HDAd5/35++ vectors; colony-forming cell (CFC) assay; detection of cell surface markers by flow cytometry; detection of human γ-globin expression by intracellular staining; globin HPLC; measurement of VCN; real-time reverse transcription PCR; cytometric bead array; anti-transgene product antibody ELISA; integration site analysis; RNA-seq analysis; and statistical analyses. A list of antibodies used for detection of cell surface markers by flow cytometry can be found in Figure S18.

## Supplemental information

Documents S1. Supplemental materials and methods and Figures S1–S18

Document S2. Article plus supplemental information

## Footnotes

Supplemental information can be found online at https://doi.org/10.1016/j.omtm.2021.12.003.
