# Supplementary material for: Safe and efficient in vivo hematopoietic stem cell transduction in nonhuman primates using HDAd5/35++ vectors
Source: Mol Ther Methods Clin Dev. 2021 Dec 6;24:127–41. doi: 10.1016/j.omtm.2021.12.003 (PMC8741415; doi:10.1016/j.omtm.2021.12.003)
Supplement: Documents S1. Supplemental materials and methods and Figures S1–S18 [file mmc1.pdf]

OMTM, Volume *24*

Supplemental information

Safe and efficient *in vivo* hematopoietic stem cell transduction in nonhuman primates using HDAd5/35++ vectors

Chang Li, Hongjie Wang, Sucheol Gil, Audrey Germond, Connie Fountain, Audrey Baldessari, Jiho Kim, Zhinan Liu, Aphrodite Georgakopoulou, Stefan Radtke, Tamás Raskó, Amit Pande, Christina Chiang, Eli Chin, Evangelia Yannaki, Zsuzsanna Izsvák, Thalia Papayannopoulou, Hans-Peter Kiem, and André Lieber

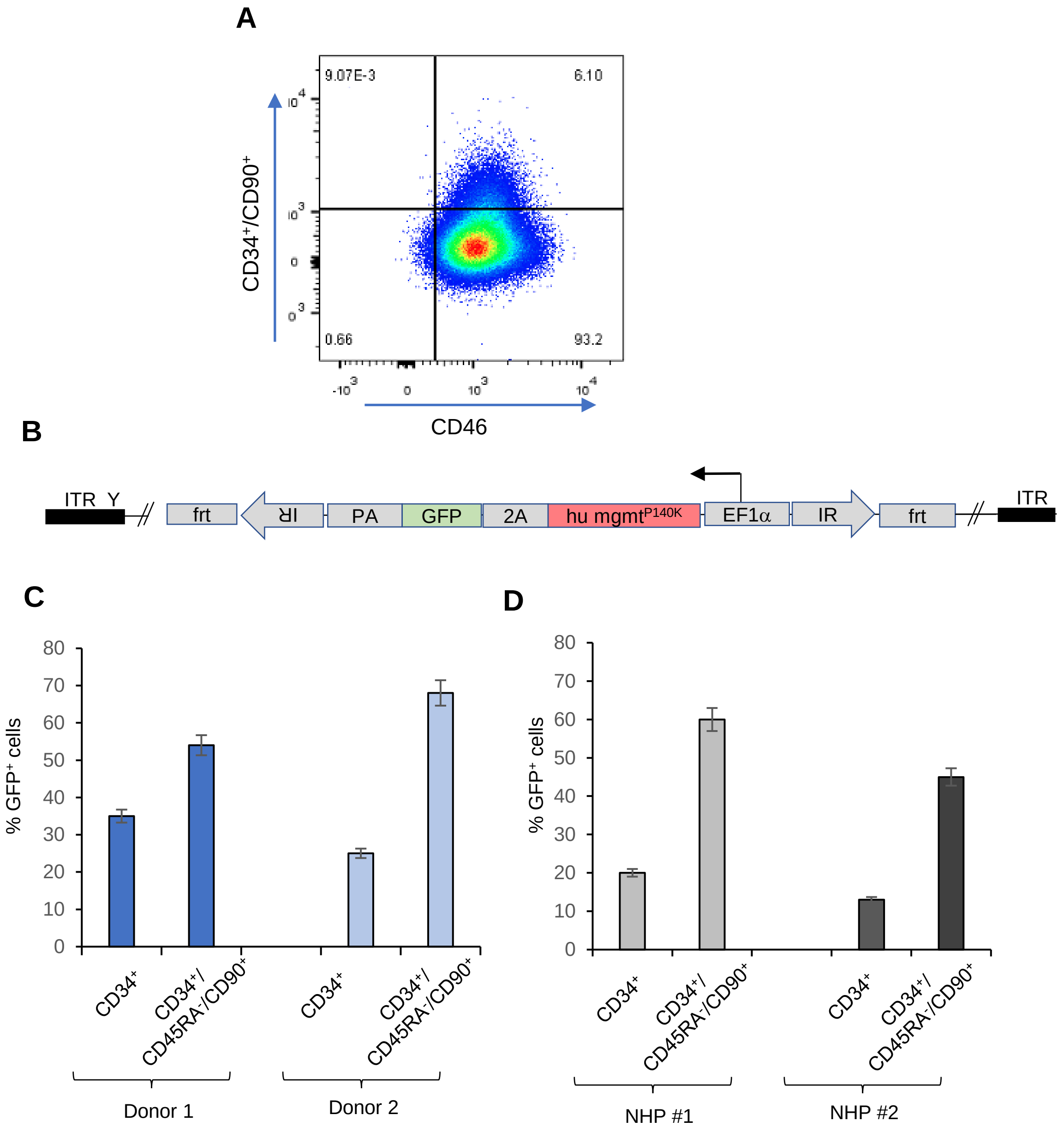

Figure S1. In vitro transduction of human and rhesus HSCs with a GFP-expressing HDAd5/35++ vector. A) CD46 expression in human $CD34^+CD90^+CD45RA^-$ cells. Representative flow cytometry. **B)** Structure of HDAd5/35++ vector expressing GFP under the control of the EF1α promoter. **C) and D)** HDAd5/35++ transduction of human (C) and rhesus (D) $CD34^+$ cells. At day 3 after infection at an MOI of 3000vp/cell, cells were stained with CD34, CD90 and CD45RA antibodies and the percentage of GFP-positive cells was measured by flow cytometry. Two human and rhesus donors for each transduction. N=3 (technical replicates).

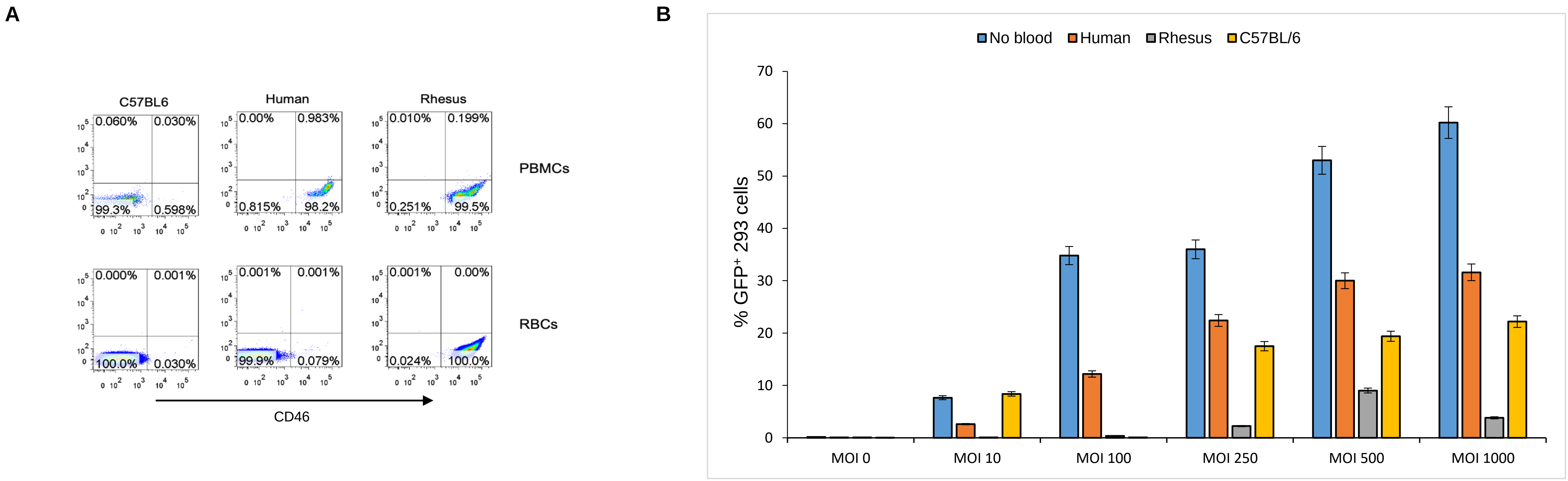

Figure S2. Sequestration of HDAd5/35++ by CD46 present on rhesus erythrocytes. A) Flow cytometry for CD46 on PBMCs and red blood cells (RBCs) from different species: C57Bl/6 mice, humans, and rhesus macaques. Flow cytometry confirmed that CD46 is expressed on human and rhesus PBMCs. It further confirms the absence of CD46 expression in human RBCs that is in contrast to the high CD46 expression found in rhesus RBCs. Representative samples are shown. **B)** Inhibition of Ad5/35++ transduction of 293 test cells by RBCs from different species. 100µl EDTA-blood was washed with 10ml PBS and resuspended in 600µl DMEM/FCS and 100µl was added to 293 cells in 96 well plates together with 100µl of HDAd5/35++-GFP containing medium for one hour under shaking. The RBC/Ad mixture was then removed, cells were washed, and GFP was measured 48 hours later. N=3

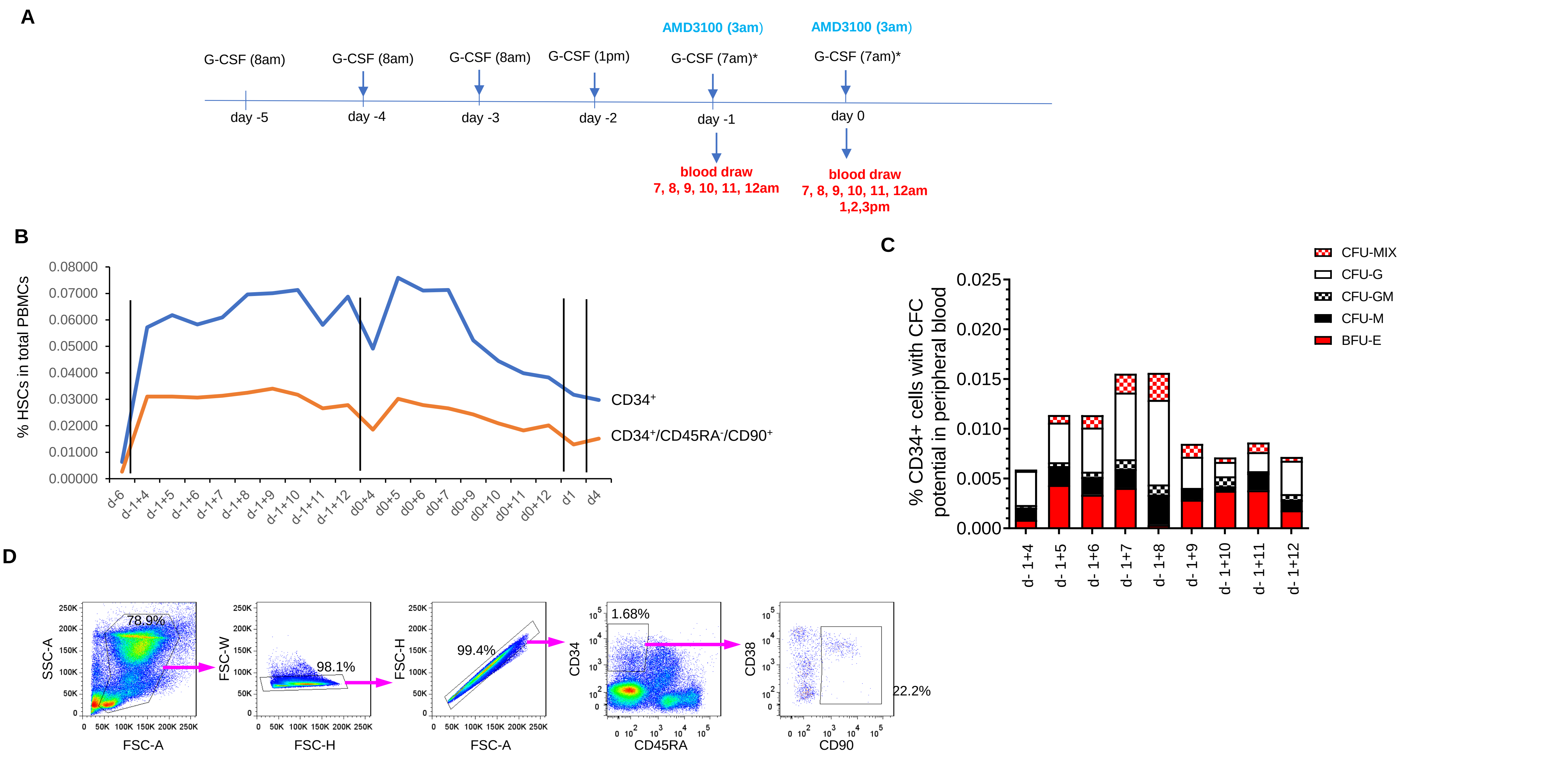

Figure S3. HSC mobilization kinetics. A) Scheme of experiment. A rhesus macaque was mobilized with G-CSF and AMD3100 at the indicated time points. Blood was drawn 4, 5, 6, 7, 8, 9, 10, and 11 hours after the AMD3100 injection. **B)** The percentages of $CD34^{+}$ and $CD34^{+}CD45RA^{-}/CD90^{+}$ were measured by flow cytometry. d-1+4 means "day -1, 4 hours after AMD3100", etc. The regimen was designed to induce two "waves" of mobilization allowing for two rounds of HDAd injection. **C)** $CD34^{+}$ cells were plated for colony formation and the type and number of colonies were assessed 12 days after plating. Shown are data for day -1. **D)** Gating strategy for $CD34^{+}CD45RA^{-}/CD90^{+}$ cells.

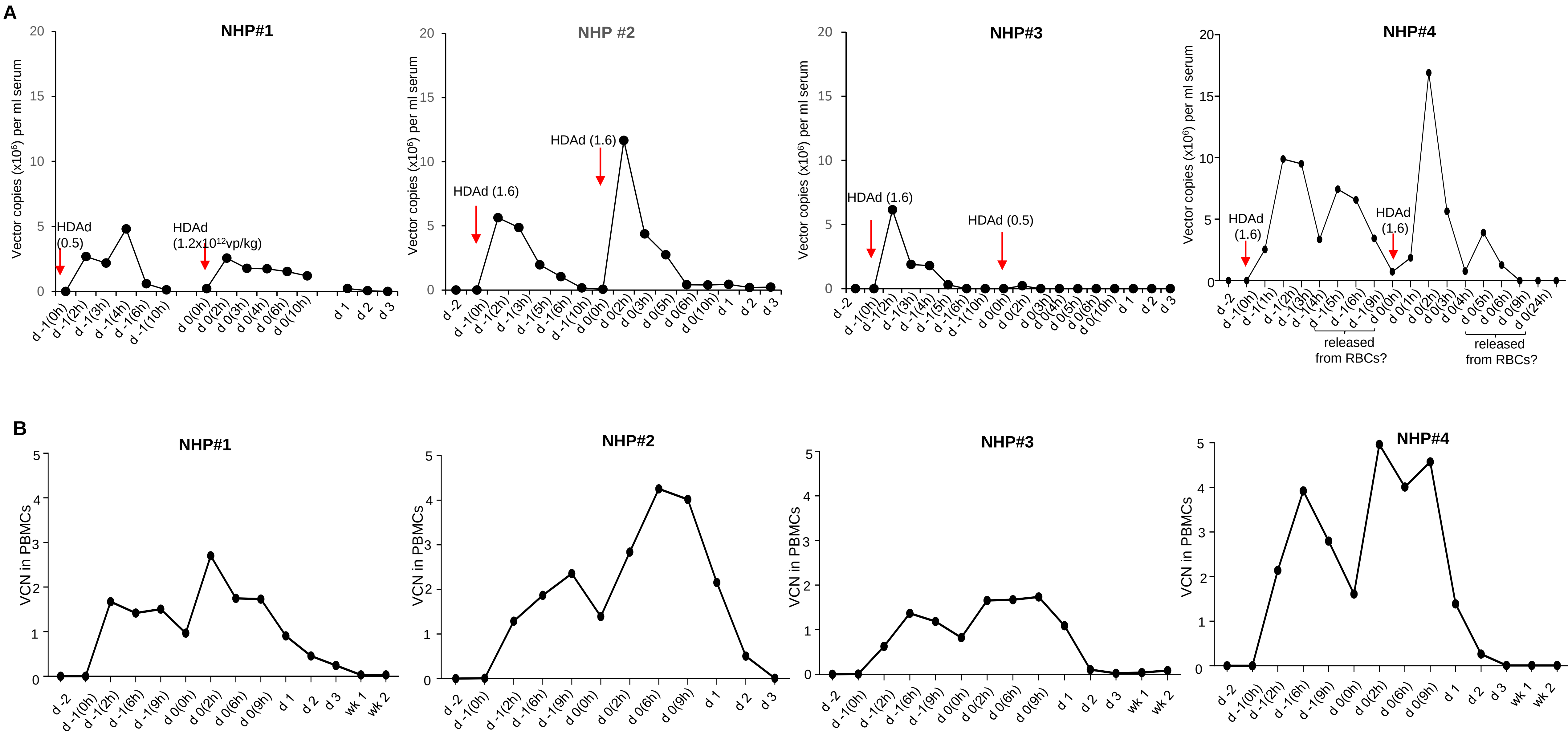

Figure S4. Vector clearance from blood. A) HDAd vector genome copies in serum samples were measured by qPCR. The long shoulder and second peak (pronounced in NHP#4) could be due to the release of HDAd5/35++ particles from RBCs. **B)** HDAd genomes in PBMCs reflecting HDAd vector binding and transduction. Shown is the vector copy number (VCN) per cell

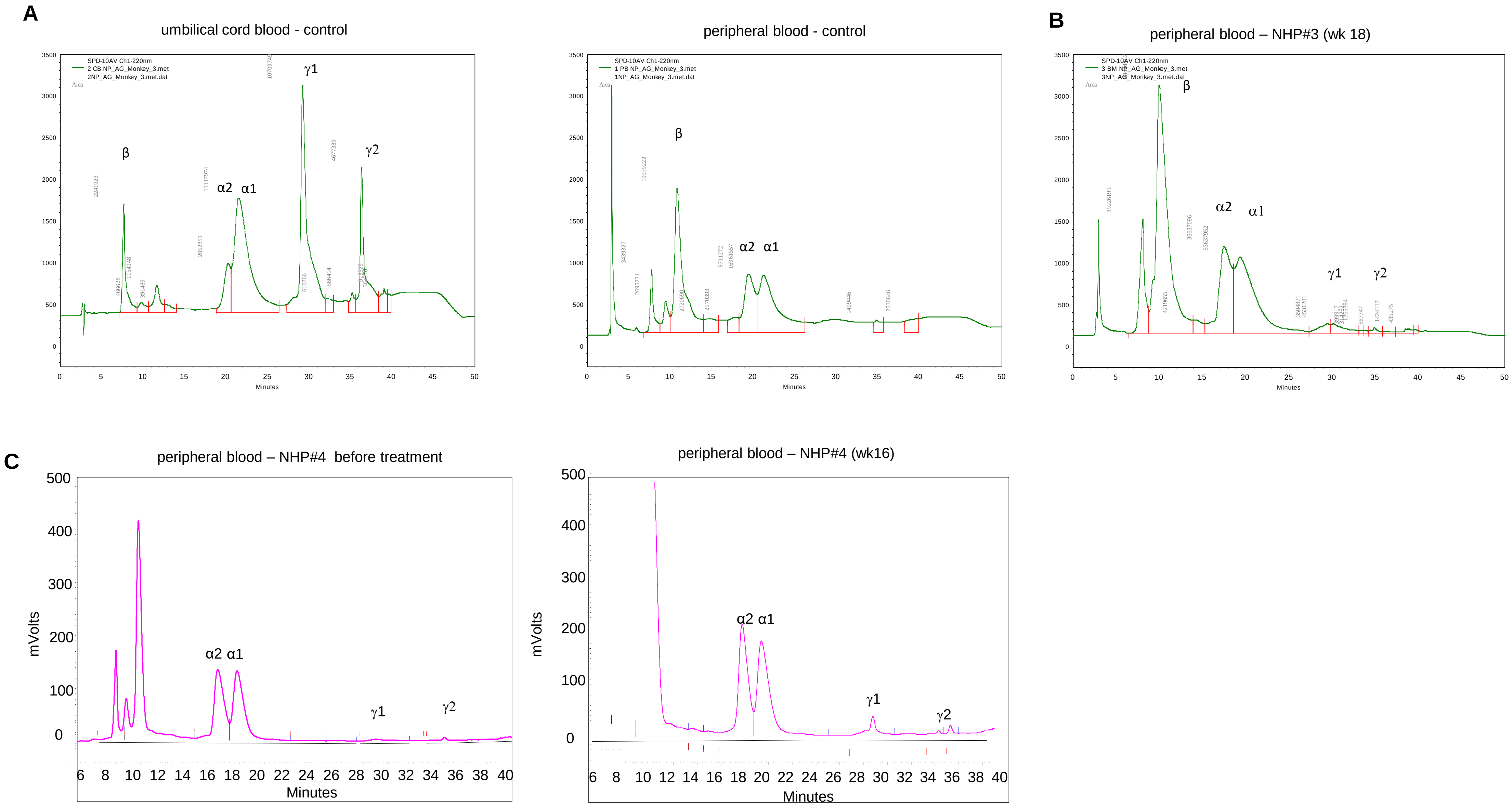

Figure S5. HPLC chromatograms. Rhesus cord (fetal) blood and peripheral blood from an untreated rhesus macaque was analyzed for globin chains. Note the localization of fetal γ1 and γ2 globin. The percentage of total monkey γ-globin to monkey α1-globin was 0.12% and 44.5% in rhesus adult and cord blood, respectively. **B, C)** Representative NHP#3 and 4 HPLC data.

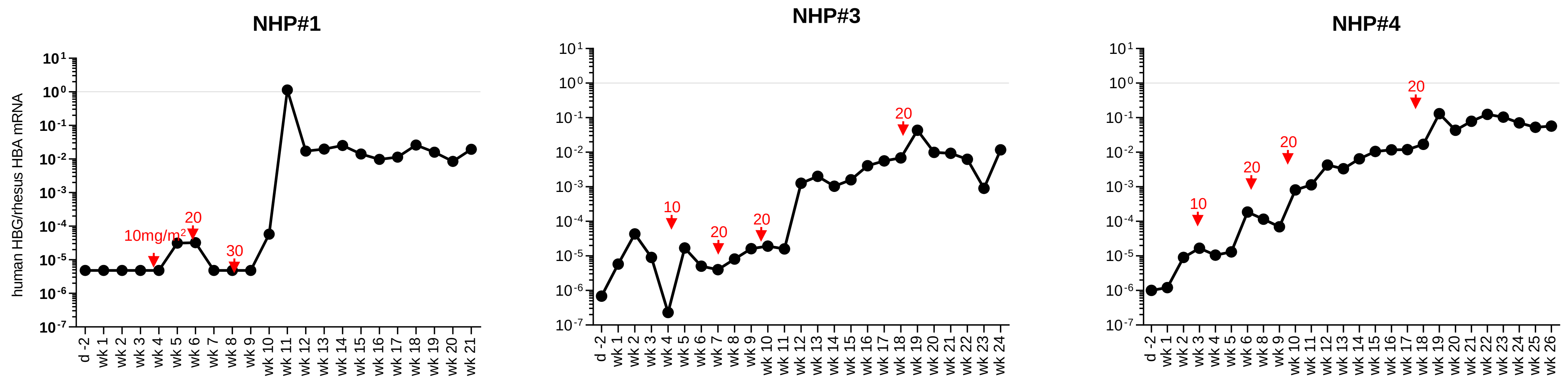

Figure S6. HBG mRNA in total peripheral blood cells. Human γ-globin were measured by qRT-PCR in comparison to rhesus α-globin mRNA. *In vivo* selection is indicated by red arrows. Note that there are ~1000-fold more RBCs in peripheral blood than PBMCs. Therefore, the numbers reflect mRNA in RBCs.

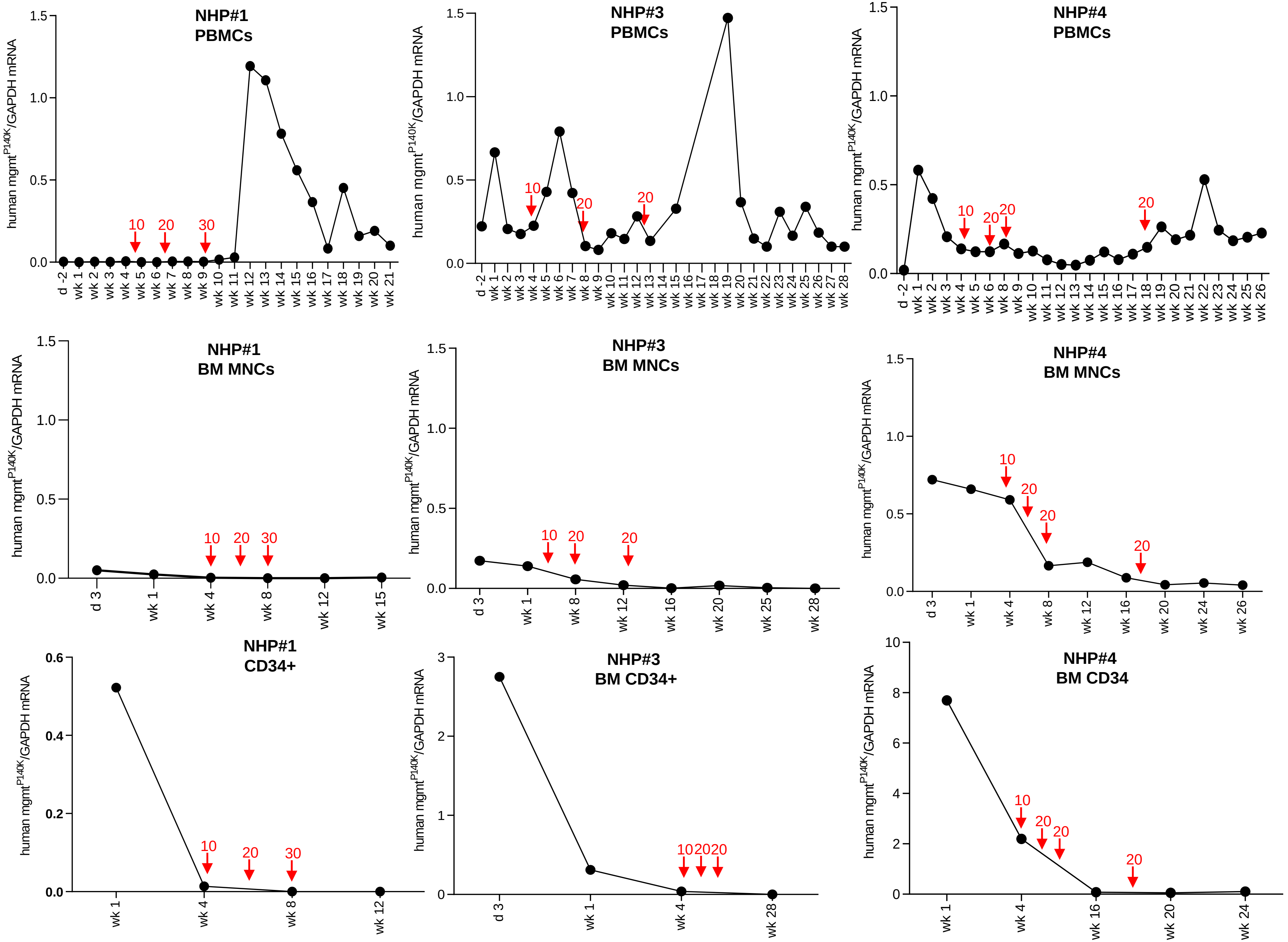

Figure S7. Percentage of mgmt^{P140K} mRNA relative to GAPDH mRNA in PBMCs, bone marrow mononuclear cells (BM MNCs) and bone marrow CD34^{+} cells. *In vivo* selection is indicated by red arrows.

A

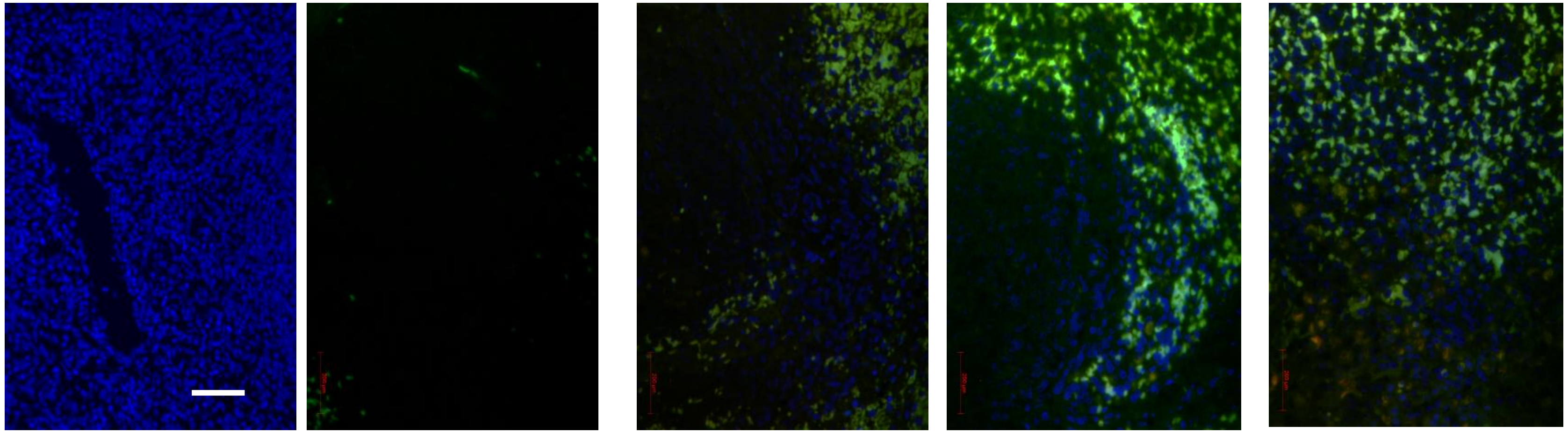

Control monkey (spleen) DAPI - blue | Control monkey (spleen) hu MGMT - green | NHP#1 spleen (hu MGMT - green) | NHP#3 spleen (hu MGMT - green) | NHP#4 spleen (hu MGMT - green)

B

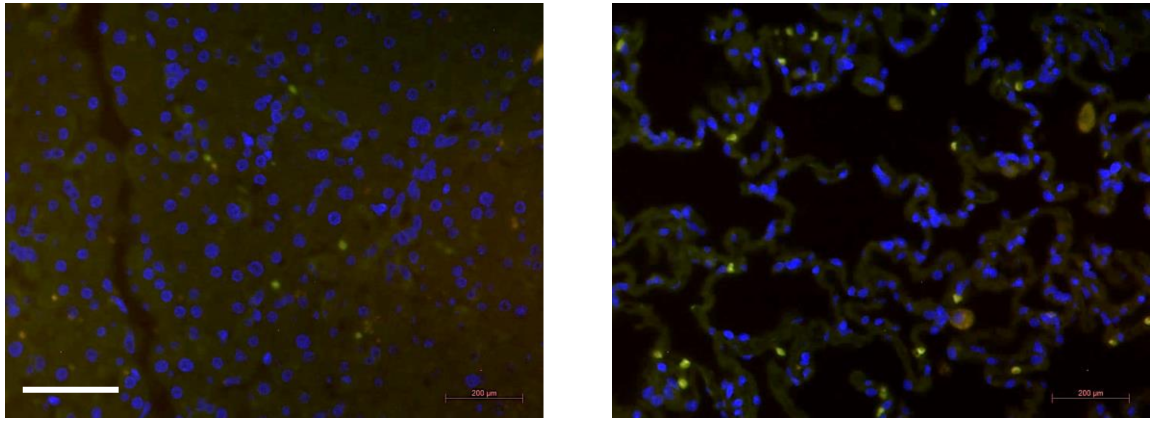

NHP#3 liver (hu MGMT - green) | NHP#3 lung (hu MGMT - green)

Figure S8. MGMT immunofluorescence staining on tissues sections. A) spleen. The left two panels show control staining. In spleen sections for NHP#1, 3,and 4, note that stained cells are predominantly in the red pulp and not in the germinal center. The scale bar is 20 μm. **B)** Liver and lung sections from NHP#3 stained with antibodies against human MGMT (green). The scale bar is 20 μm.

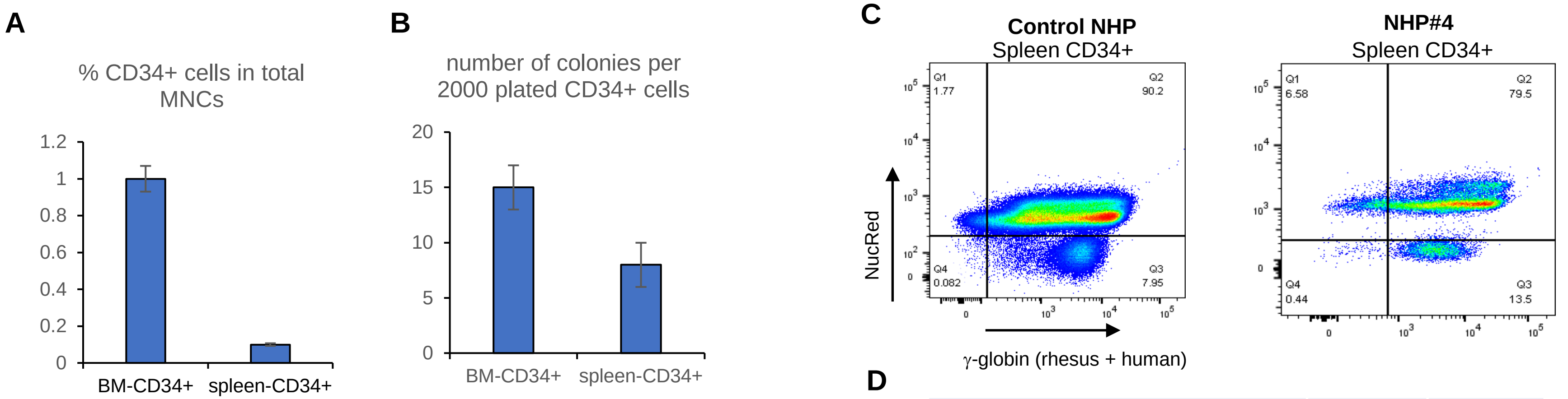

D

	Control NHP	NHP#4
% γ-globin⁺ in nucleated EBs	90.2	79.5
% γ-globin⁺ in enucleated EBs	7.95	13.6
γ-globin MFI in nucleated EBs	2606	8129
γ-globin MFI enucleated	2118	3489
Human HBG/rhesus HBA mRNA	0	1.18E-04

Figure S9. Hematopoietic potential of splenic CD34⁺ cells and human γ-globin expression after *in vitro* erythroid differentiation. A) Percentage of CD34⁺ cells in bone marrow and spleen of NHP#4 at necropsy. **B)** Number of progenitor colonies derived from bone marrow and spleen CD34⁺ cells of NHP#4. The number of colonies was counted 12 days after plating of 2000 CD34⁺ cells. The distribution of CFU-mix, CFU-G, CFU-GM and CFU-E colonies was similar for spleen and bone marrow CD34⁺ cells. **C, D)** *In vitro* erythroid differentiation (ED) of splenic CD34⁺ cells from an untreated rhesus macaque and NHP#4 at 6 months after HDAd5/35++ injection. **C)** Flow cytometry for γ-globin and Nuclear Red at day 13 of ED. Note that at this stage erythroblasts express rhesus HBA and HBF and that the anti-γ-globin antibody reacts with both added human and endogenous rhesus γ-globin. **D)** Summary of flow and globin mRNA data in the control animal and NHP#4. The qRT-PCR primers selectively measure human γ-globin mRNA.

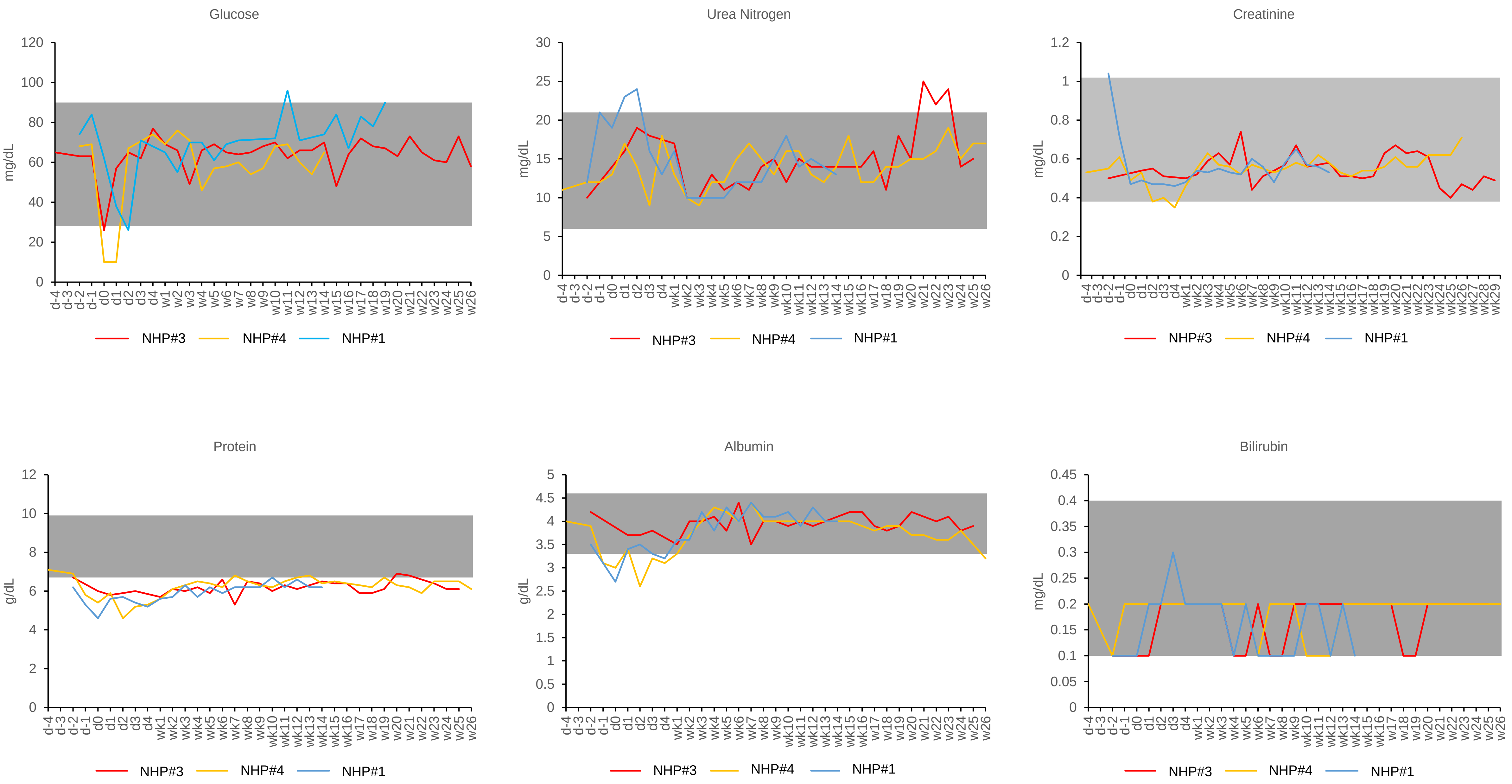

Figure S10. Selected hematological parameters (part 1)

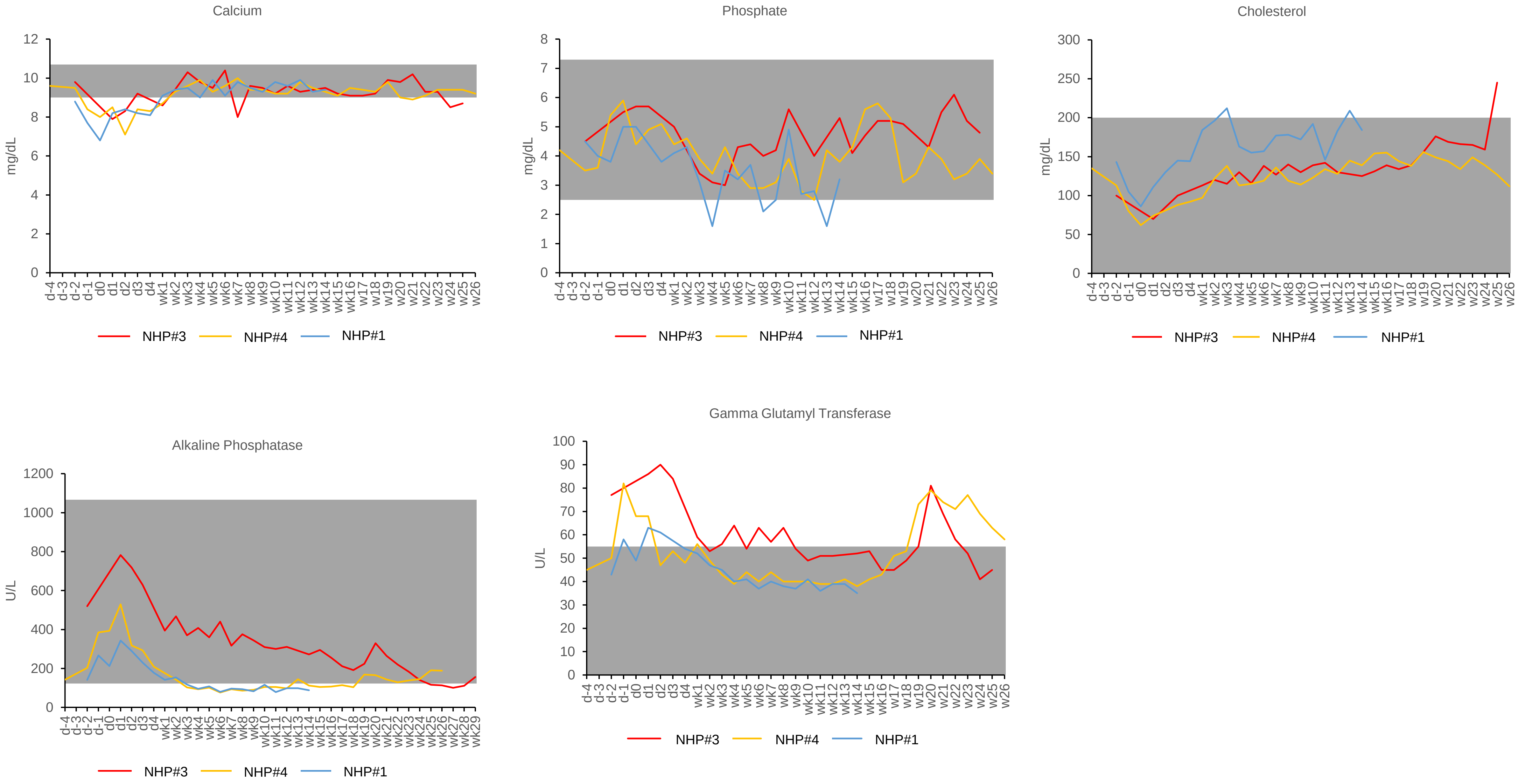

Figure S10. Selected hematological parameters (part 2)

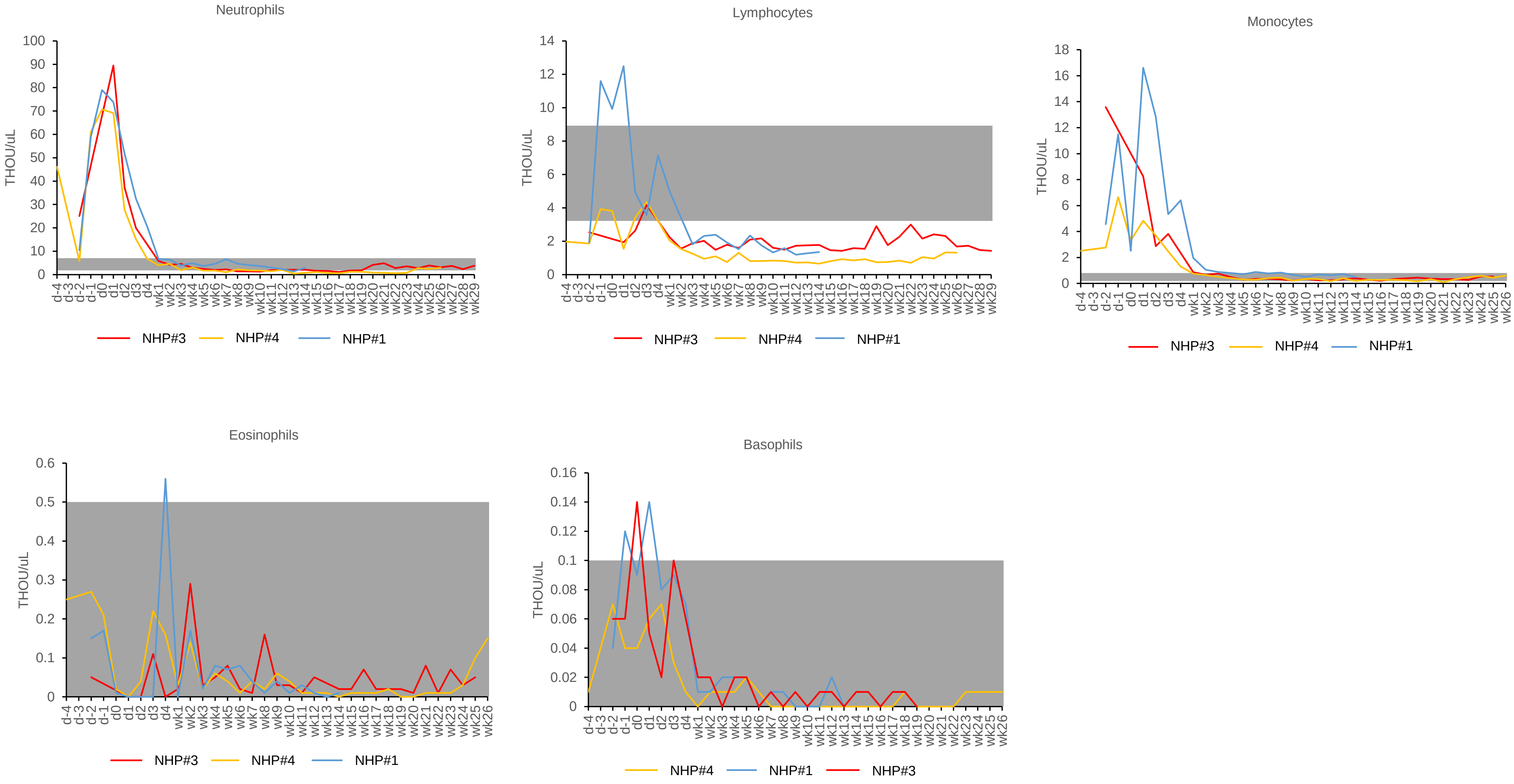

Figure S10. Selected hematological parameters (part 3).

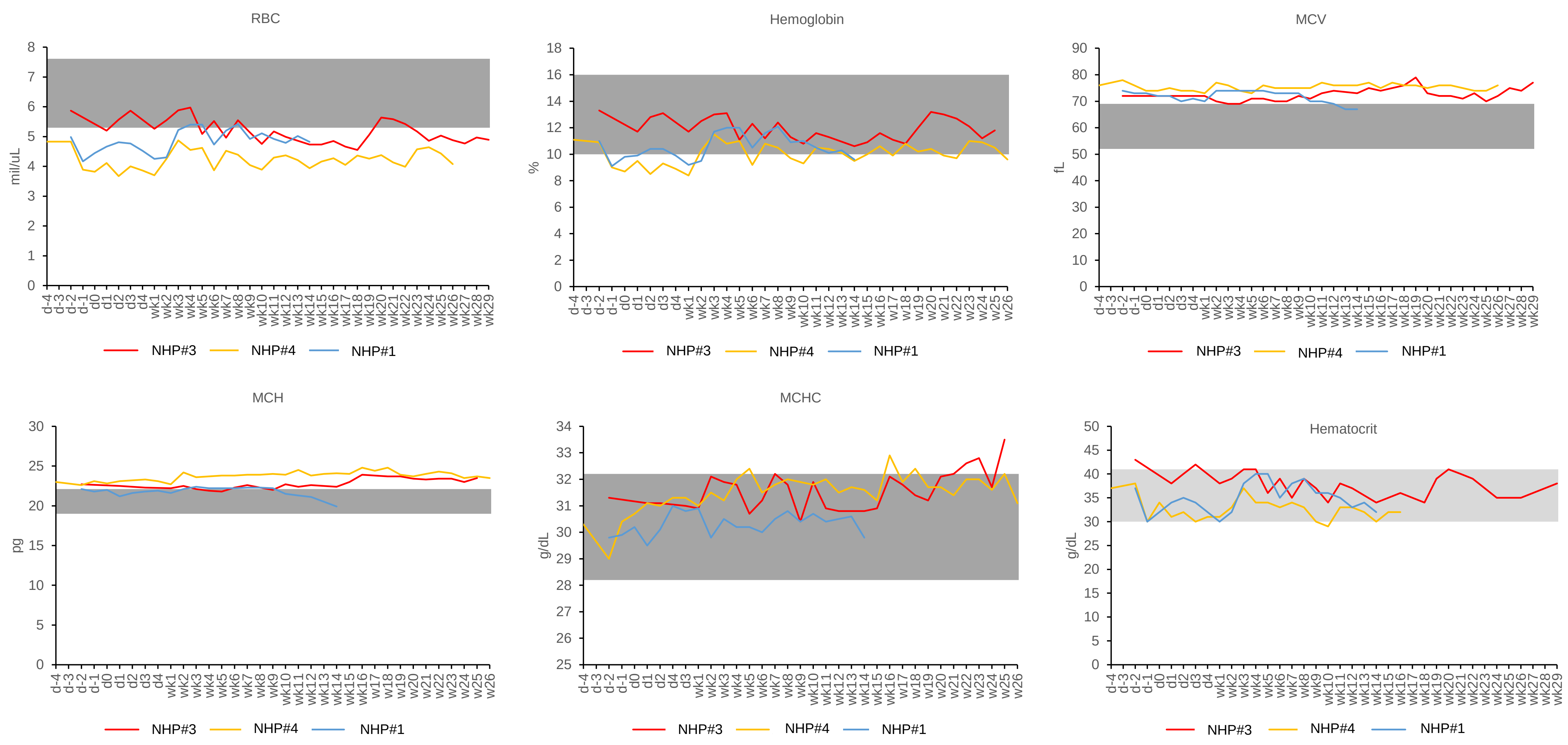

Figure S10. Selected hematological parameters (part 4). MCH: Mean Corpuscular Hemoglobin, MCHC: Mean Corpuscular Hemoglobin Concentration.

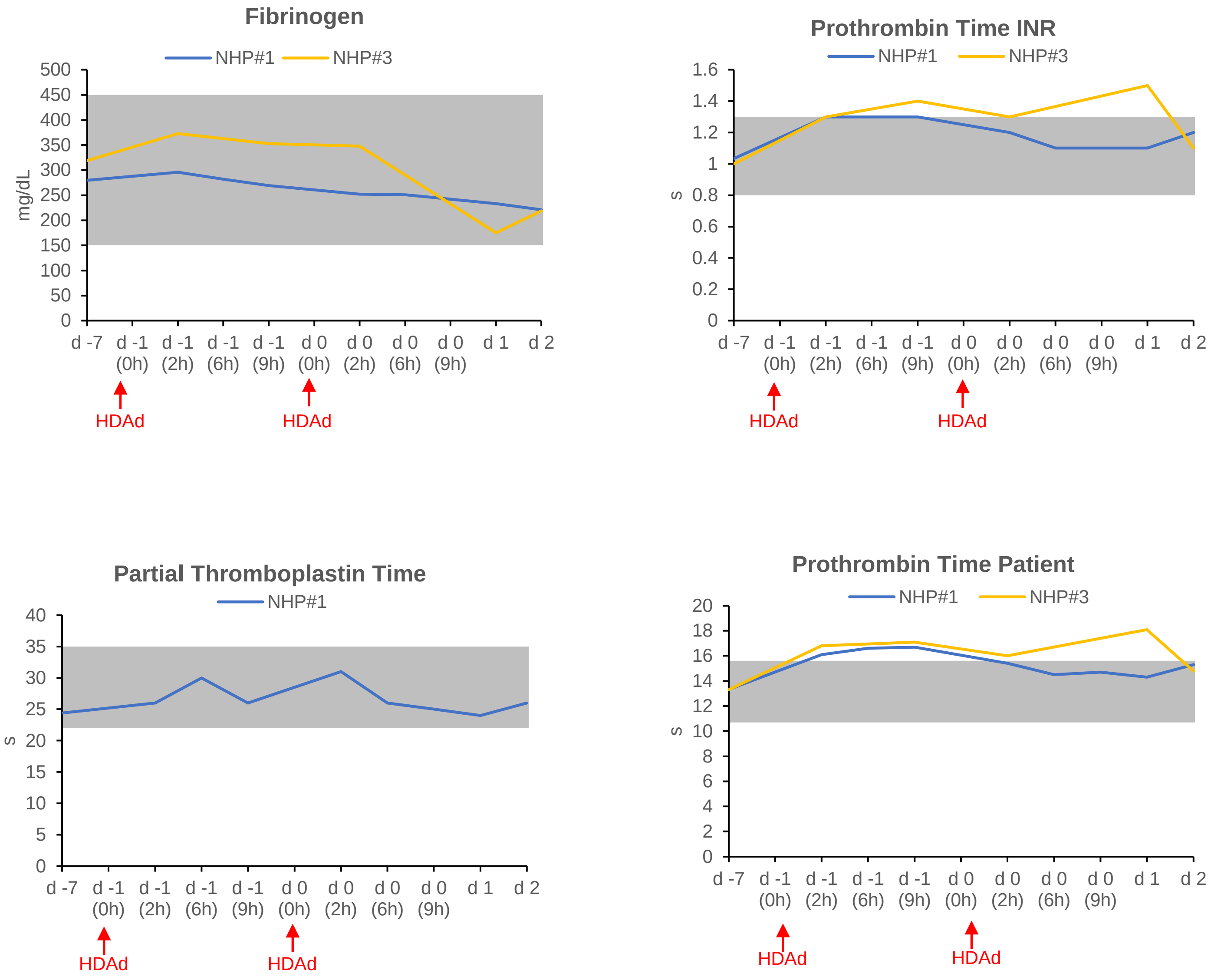

Figure S10. Complement and coagulation parameters (part 5)

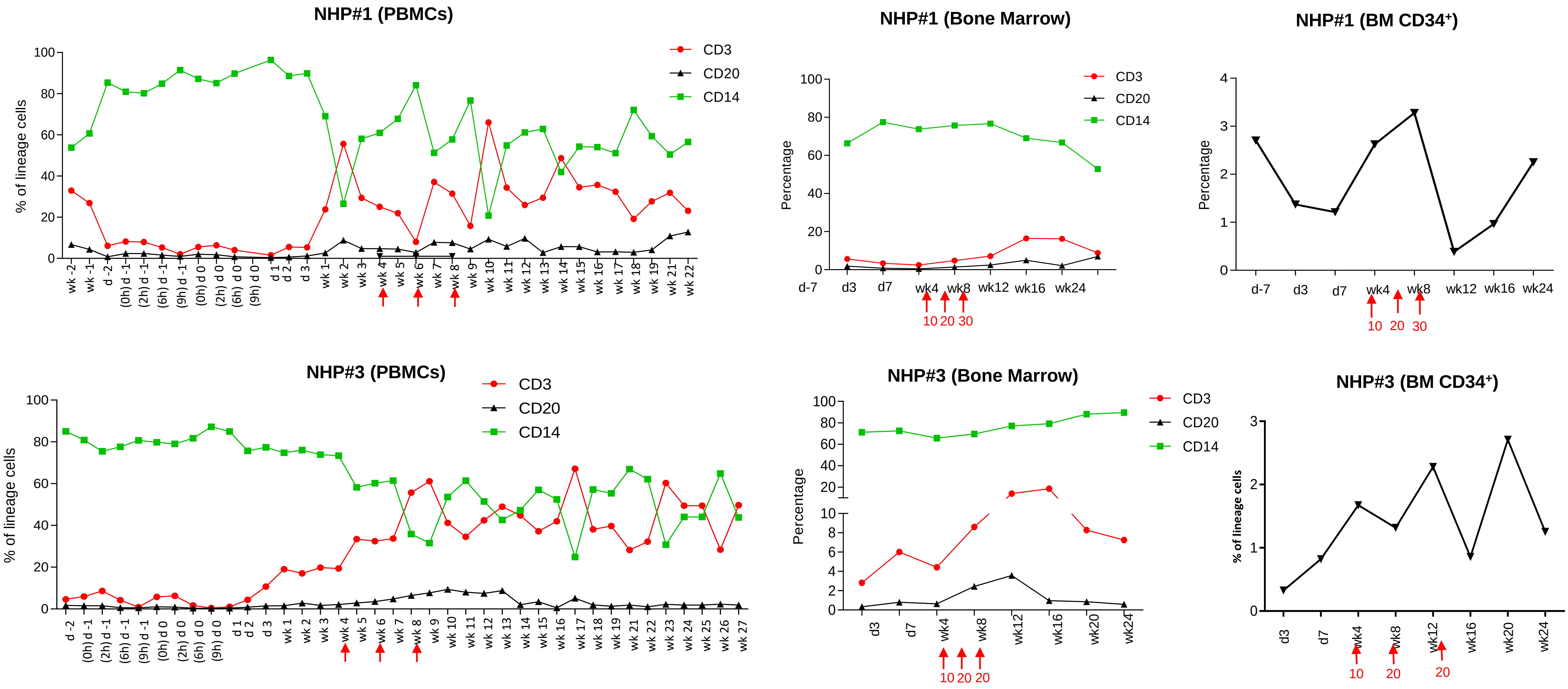

Figure S11. Lineage-positive cells in PBMCs and bone marrow mononuclear cells for NHP#1, and 3. CD3$^-$, CD20$^-$, CD14$^-$, and CD34$^+$ positive cells were measured by flow cytometry. Shown is the percentage of a given lineage in all PBMCs or BM MNCs.

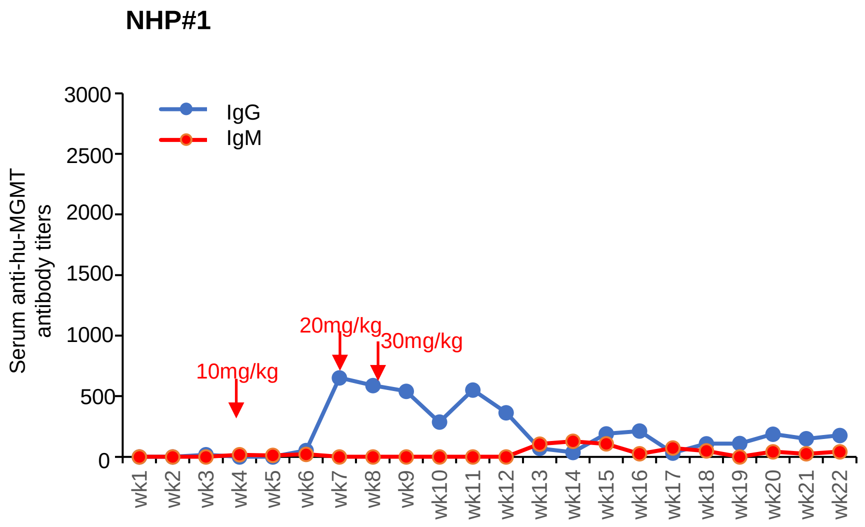

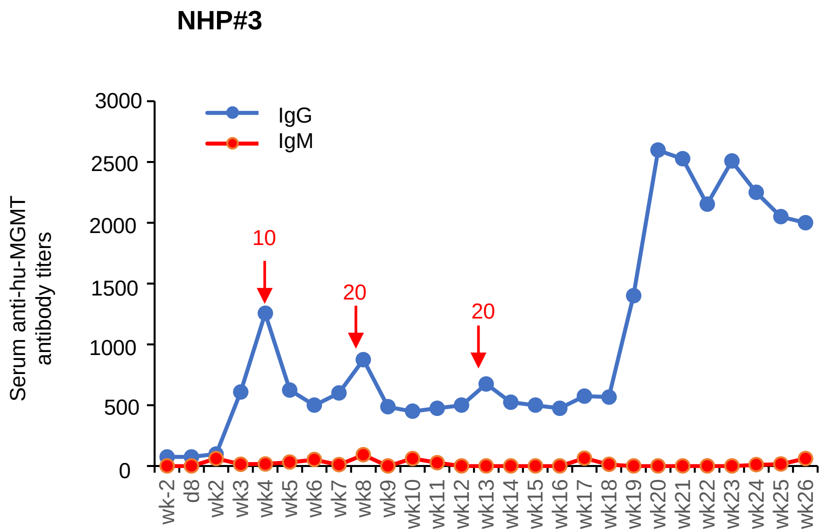

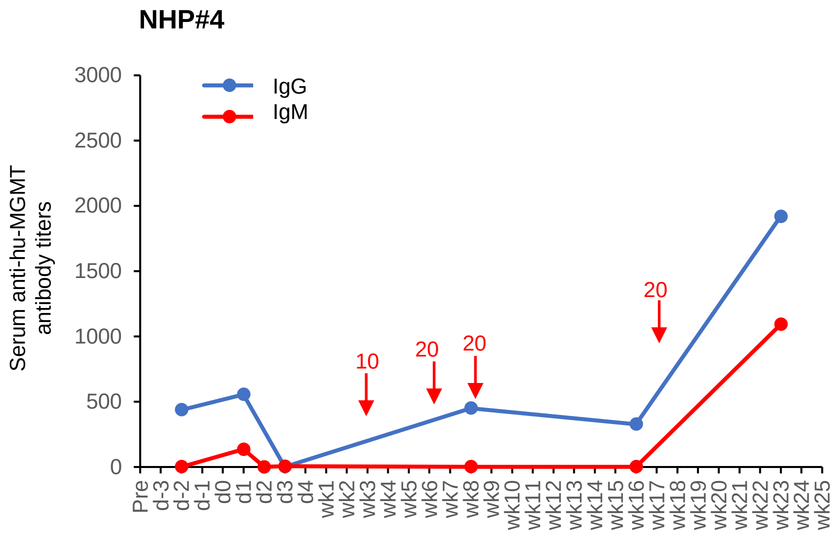

Figure S12. Serum IgG and IgM antibodies against the human MGMT

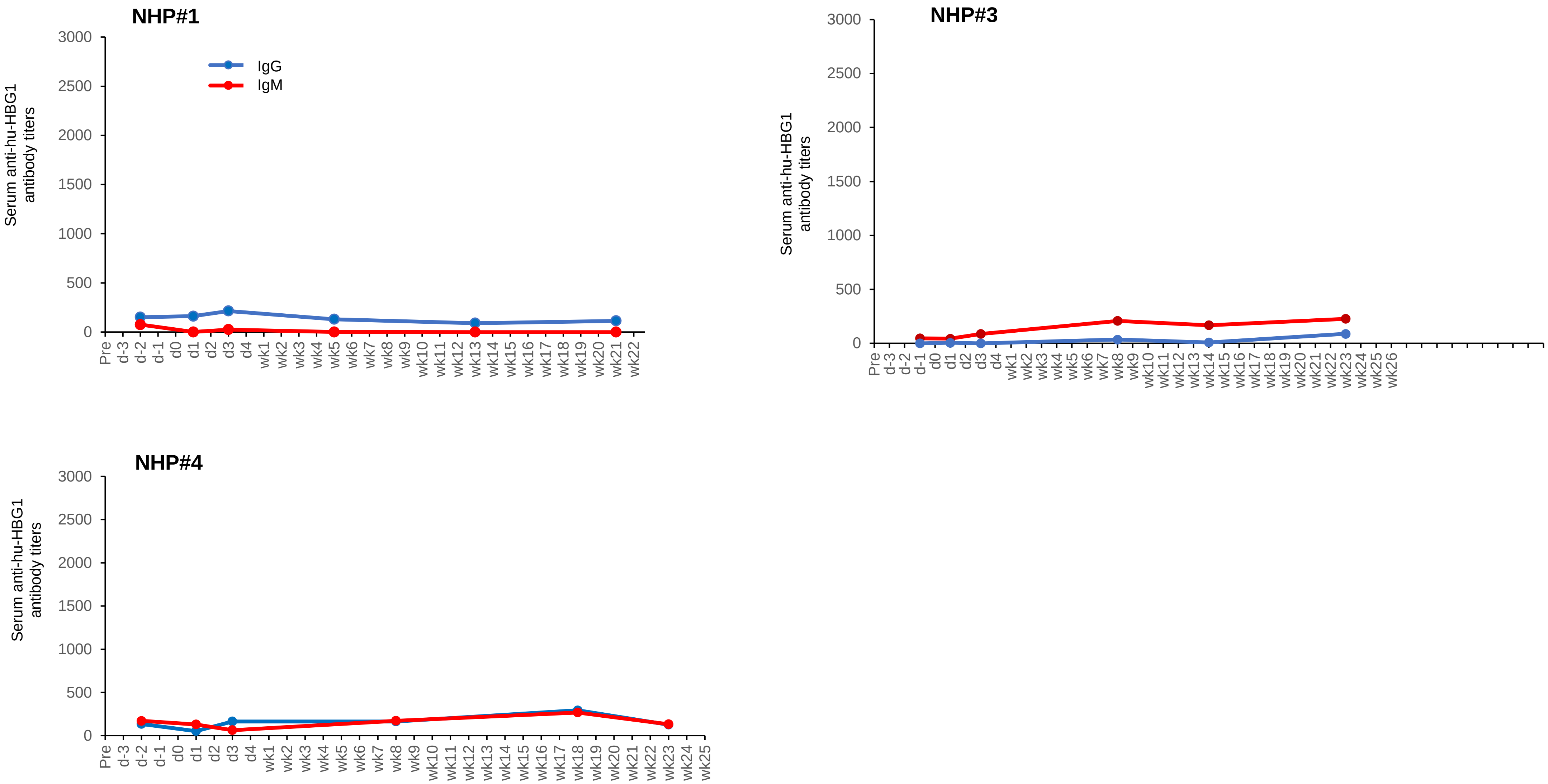

Figure S13. Serum IgG and IgM antibodies against the human γ-globin

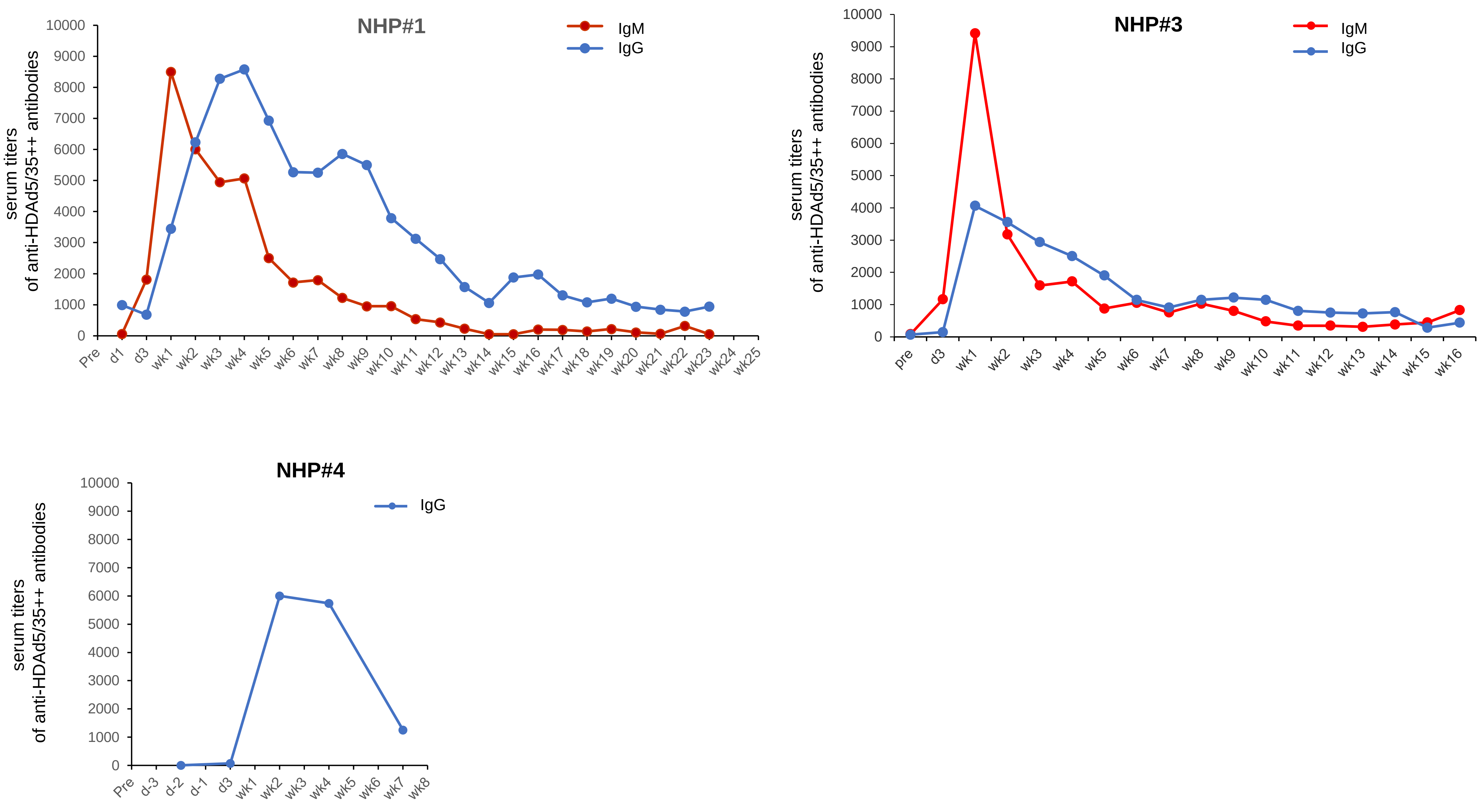

Figure S14. Serum IgG and IgM antibodies against the HDAd5/35++ capsid

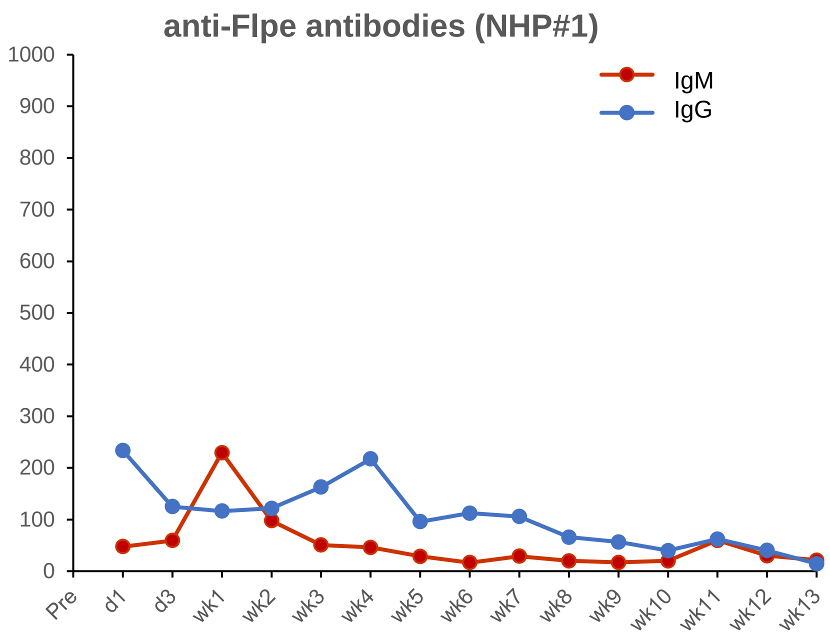

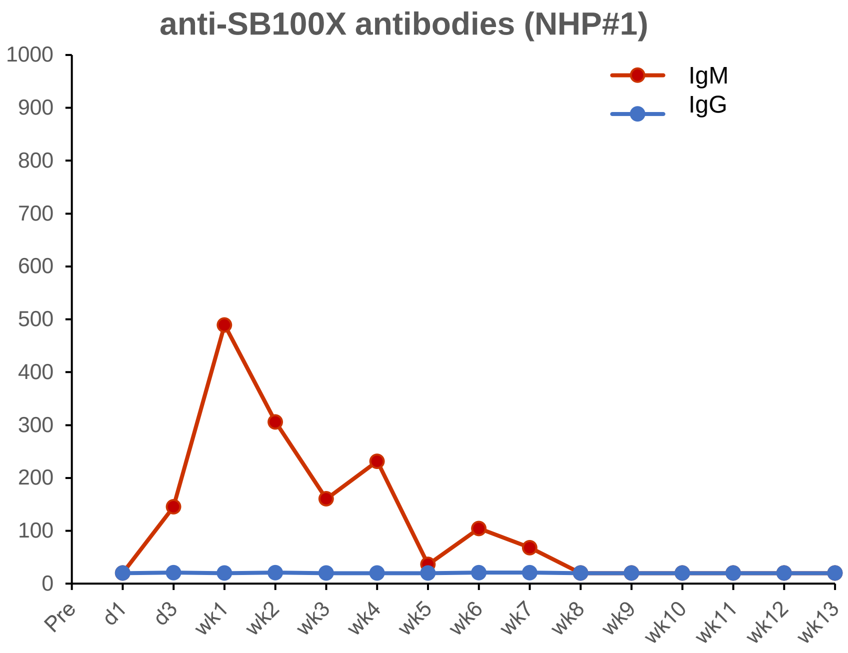

Figure S15. Serum IgG and IgM antibodies against the editing enzymes (Flpe, SB100x) measured in NHP#1

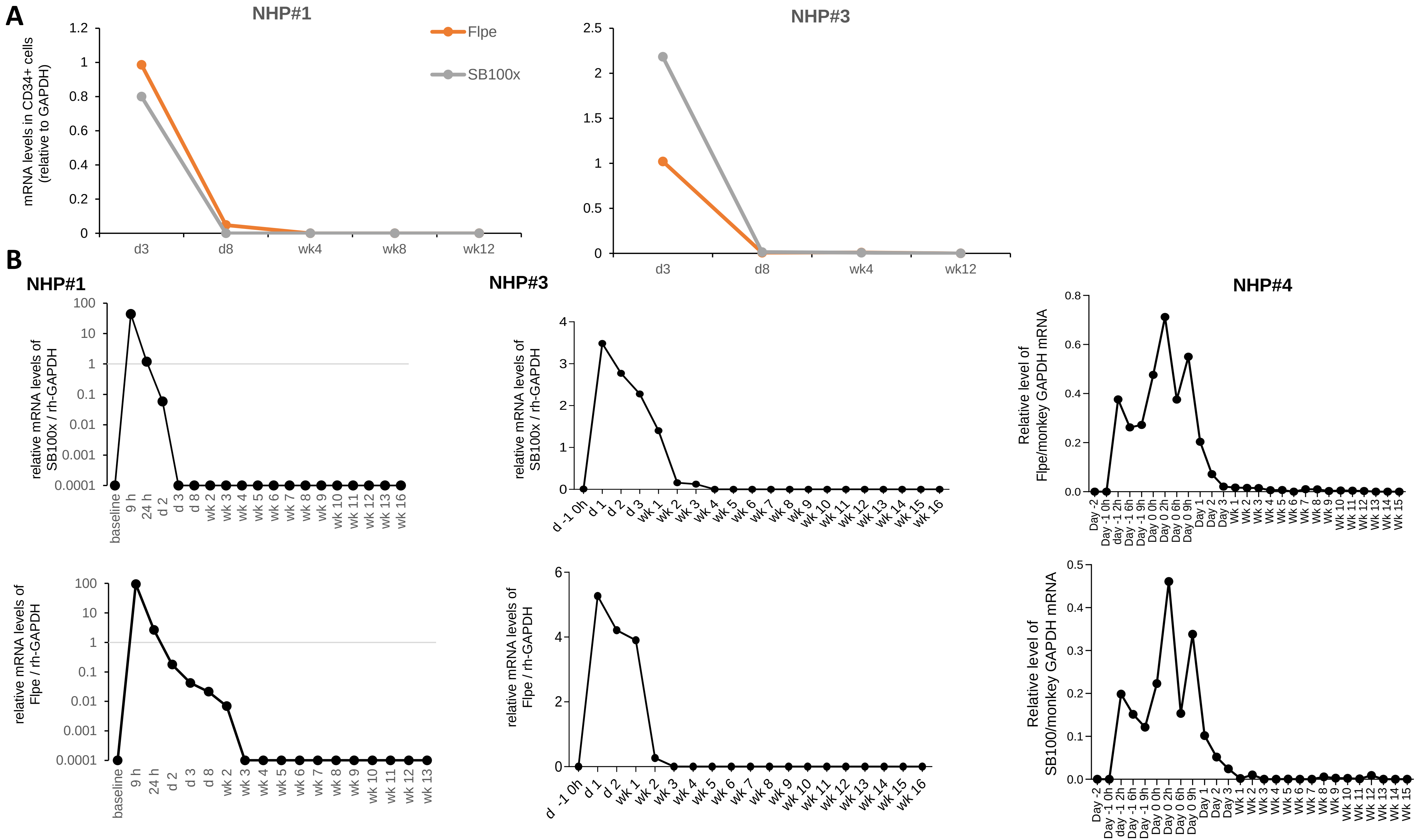

Figure S16. mRNA expression of editing enzymes in NHP#1 and 3. A) mRNA levels in bone marrow CD34^{+} cells relative to GAPDH mRNA levels. **B)** mRNA levels in PBMCs.

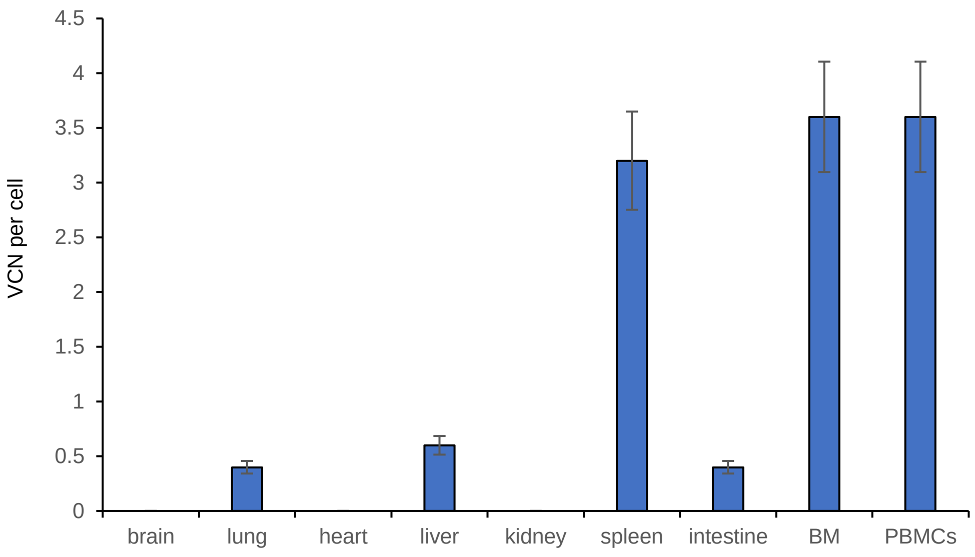

Figure S17. HDAd5/35++ vector genome biodistribution (VCN per cell) in CD46-transgenic mice at week 16 after mobilization and *in vivo* transduction/selection (week 16).

CD34-PE	PE anti-human CD34 Antibody	2µL	343606	561	BioLegend	Mouse IgG2a, κ
CD38-APC	CD38-APC	2µL	100845	OKT10	Caprico Bio	Mouse IgG1, k
CD90-BV421	Brilliant Violet 421™ anti-human CD90 (Thy1) Antibody	2µL	328122	5E10	BioLegend	Mouse IgG1, κ
CD45RA-PE/Cy7	PE-Cy™7 Mouse Anti-Human CD45RA	2µL	561216	5H9	BD Biosciences	Mouse IgG1, κ
CD3-APC/Cy7	APC-Cy™7 Mouse Anti-Human CD3	2µL	557757	SP34-2	BD Biosciences	Mouse BALB/c IgG1, λ
CD4-BV510	Brilliant Violet 510™ anti-human CD4 Antibody	2µL	317444	OKT4	BioLegend	Mouse IgG2b, κ
CD8-BV421	Brilliant Violet 421™ anti-human CD8a Antibody	2µL	301036	RPA-T8	BioLegend	Mouse IgG1, κ
CD25-APC	Alexa Fluor 647 anti-human CD25 Antibody	2µL	302618	BC96	BioLegend	Mouse IgG1, κ
CCR7-PE	PE Mouse anti-Human CD197	7.5µL	560765	150503	BD Biosciences	Mouse IgG2a
CD95-APC	APC anti-human CD95 (Fas) Antibody	2µL	305612	DX2	BioLegend	Mouse IgG1, κ
CD28-BV605	Brilliant Violet 605™ anti-human CD28 Antibody	2µL	302968	CD28.2	BioLegend	Mouse IgG1, κ
CXCR3-PerCP/Cy5.5	PerCP-Cy™5.5 Mouse Anti-Human CD183	2µL	560832	1C6/CXCR3	BD Biosciences	Mouse BALB/c IgG1, κ
CD45-APC	CD45-APC, non-human primate	3µL	130-091-900	MB4-6D6	Miltenyi	mouse IgG1κ
CD14-BV421	Brilliant Violet 421™ anti-human CD14 Antibody	2µL	301830	M5E2	BioLegend	Mouse IgG2a, κ
CD20-PE/Cy7	PE/Cy7 anti-human CD20 Antibody	2µL	302312	2H7	BioLegend	Mouse IgG2b, κ
γ-PE	Hemoglobin γ Antibody (51-7): sc-21756	3µL	sc-21756 PE	51-7	Santa Cruz	mouse IgG1κ
NucRed-647	NucRed™ Live 647 ReadyProbes™ Reagent	1 drops/mL	R37106		ThermoFisher Scientific	
CD71-BV510	BD OptiBuild™ BV510 Mouse Anti-Human CD71	2µL	744926	L01.1	BD Biosciences	Mouse BALB/c IgG2a
Fc Blocker	Human BD Fc Block™	5µL (2.5µg)	564220	ND	BD Pharmingen™	ND
CD46	CD46, Human, mAb M177	3µL	HM2103	M177	Hycult Biotech	Mouse IgG1
anti-mouse IgG	R-Phycoerythrin AffiniPure $F(ab')_2$ Fragment Goat Anti-Mouse IgG (H+L)	1µL	115-116-146	Polyclonal	Jackson ImmunoResearch	$F(ab')_2$ Fragment

Fig.S18. Flow cytometry antibodies

Suppl. Materials and Methods

HDAd5/35++ vectors. HDAd-SB, HDAd-mgmt/GFP, HDAd-γ-globin-hu-mgmt^{P140K} and HDAd-long-LCR-γ-globin-hu-mgmt^{P140K} have been described earlier [1-3]. For the production of HDAd5/35++ vectors, corresponding plasmids were linearized and rescued in 116 cells [4] with AdNG163-5/35++, an Ad5/35++ helper vector containing chimeric fibers composed of the Ad5 fiber tail, the Ad35 fiber shaft, and the affinity-enhanced Ad35++ fiber knob [5]. HD-Ad5/35++ vectors were amplified in 116 cells cultured in 3-liter spinner flasks as described in detail elsewhere [4]. After collection from the CsCl gradient, HDAd5/35++ vectors were dialyzed against a total of 2 liters of the following 25mM Tris.pH 7.5, 140mM NaCl, 5mM KCl, 0.6mM Na_2HPO_4, 0.5mM $MgCl_2$, 0.9mM $CaCl_2$, 5% sucrose and stored for less than two months before being thawed for infusion. Helper virus contamination levels were found to be <0.05%. Titers were 3-8x10^{12} viral particles (vp/ml.

CD34$^+$ cell culture: Human CD34$^+$ cells from G-CSF-mobilized adult donors were recovered from frozen stocks and incubated overnight in Iscove's modified Dulbecco's medium (IMDM) supplemented with 10% heat-inactivated FCS, 1% BSA 0.1 mmol/l 2-mercaptoethanol, 4 mmol/l glutamine and penicillin/streptomycin, Flt3 ligand (Flt3L, 25 ng/ml), interleukin 3 (10 ng/ml), thrombopoietin (TPO) (2 ng/ml), and stem cell factor (SCF) (25 ng/ml). Rhesus CD34$^+$ cells were isolated using a human CD34 MicroBead kit from Miltenyi Biotech. Flow cytometry demonstrated that >98% of cells were CD34-positive. Cytokines and growth factors were from Peprotech (Rocky Hill, NJ). CD34$^+$ cells were transduced with virus in low attachment 12 well plates.

Isolation of CD34$^+$ cells from spleen and bone marrow: Briefly, after necropsy, spleen tissue was cut into small pieces with scissors in a petri dish and was then gently and repeatedly pressed through a cell strainer with a 70 μm nylon mesh (Fisher Scientific, Cat# 22363548) using the plunger of a 3 mL syringe. The cell suspension was centrifuged for 10 min at 300 g. The supernatant was carefully removed, and the cell pellet was re-suspended by pipetting up and down gently. Erythrocytes were lysed with BD Pharm Lyse.

The cell pellet was washed with PBS and filtered using the cell strainer. The cell number and viability were determined using Trypan blue staining. Spleen and bone marrow CD34$^+$ cells were isolated using PE-anti-human CD34$^+$ antibodies (Biolegend, cat# 343606) and anti-PE microbeads (Miltenyi Biotec) by following the manufacturer's instructions.

***In vitro* erythroid differentiation of rhesus CD34$^+$ cells:** Monkey CD34$^+$ cell *in vitro* erythroid differentiation was done based on the protocol developed by Douay [6]. In brief, in step 1, cells at a density of 10^4 cells/mL were incubated for 2 days in StemSpan SFEM (StemCell Technologies) media supplemented with 10^{-6} M Dexamethasone, 10 µg/mL insulin, 150 ng/mL SCF, 10 ng/mL IL-3, 3 U/mL erythropoietin (Epo), glutamine, and pen/strep. In step 2, cells at a density of 1×10^5 cells/mL were incubated for 7 days in IMDM supplemented with 30% FBS, 10^{-6} M Dexamethasone, 10 µg/mL insulin, 330 µg/mL transferrin, 150 ng/mL SCF, 10 ng/mL IL-3, 3 U/mL erythropoietin (Epo), glutamine, and pen/strep. In step 3, cells at a density of 1×10^6 cells/mL were incubated for 2 days in IMDM supplemented with 30% FBS, 10 µg/mL insulin, 330 µg/mL transferrin, 150 ng/mL SCF, 3 U/mL erythropoietin (Epo), glutamine, and pen/strep. In step 4, cells at a density of 1×10^6 cells/mL cells were incubated for 4 days in IMDM supplemented with 30% FBS, 10 µg/mL insulin, 330 µg/mL transferrin, 3 U/mL Epo, glutamine, and pen/strep.

HDAd5/35++ vectors. For the generation of the HDAd-PT4-long-LCR-γ -globin-hu mgmt vector, the DNA fragment containing the enhanced Sleeping Beauty transposon terminal inverted repeats (PT4 IRs) (sequence were based on the plasmid PT4/HB (Addgene, #108352)) flanked by FRT sites was synthesized by Genscript (Nanjing, China) and inserted into a shuttle plasmid based on the cosmid vector pWE15 (Stratagene) (pWEAd5-PT4). pWE-Ad5-PT4 contains the Ad5 5'ITR (nucleotides 1 through 436) and 3'ITR (nucleotides 35741 through 35938). The EF1α promoter-hmgmt(p140k)-SV40pA fragment was PCR amplified from pWEAd5-SB-mgmt [1] and inserted into PacI sites of pWEAd5-PT4 between the two PT4 IRs

(pWEAd5-PT4-hmgmt). The 28.9 kb fragment containing LCR-β-γ-globin-3'HS1 was cut with SalI from pAd-long-LCR-β-γ-globin [1] and inserted into the SalI site of pWEAd5-PT4-hmgmt (pWEAd5-PT4-long LCR- γ-globin-hmgmt). The complete long-LCR-γ-globin/hmgmt cassette was flanked by PT4 IR/DR sites and FRT sites. The main difference of HDAd-PT4-long LCR- γ-globin-hmgmt and HDAd-long-LCR-β-γ-globin are modification of IR/DR sequence (T0 →T4) and deletion of cHS4 from vector. The resulting plasmids were packaged into phages using Gigapack III Plus Packaging Extract (Stratagene) and propagated. To generate the helper dependent virus, the viral genomes were released by I-CeuI digestion from the plasmid for rescue in 116 cells.

For the production of HDAd5/35++ vectors, corresponding plasmids were linearized and rescued in 116 cells[4] with AdNG163-5/35++, an Ad5/35++ helper vector containing chimeric fibers composed of the Ad5 fiber tail, the Ad35 fiber shaft, and the affinity-enhanced Ad35++ fiber knob [5]. HD-Ad5/35++ vectors were amplified in 116 cells cultured in 3-liter spinner flasks as described in detail elsewhere [4]. After collection from the CsCl gradient, HDAd5/35++ vectors were dialyzed against a total of 2 liters of the following 25mM Tris.pH 7.5, 140mM NaCl, 5mM KCl, 0.6mM Na_2HPO_4, 0.5mM $MgCl_2$, 0.9mM $CaCl_2$, 5% sucrose and stored for less than two months before being thawed for infusion. Helper virus contamination levels were found to be <0.05%. Titers were 3-8x10^{12} viral particles (vp/ml.

Colony-Forming Cell (CFC) Assay. A total of 2000 sort-purified $CD34^+$ cells and CD34-subpopulations were seeded into 3.5 ml ColonyGEL 1402 (ReachBio, Seattle, WA). Colonies were scored after 12 to 14 days, discriminating colony forming unit- (CFU-) granulocyte (CFU-G), macrophage (CFU-M), granulocyte-macrophage (CFU-GM) and burst forming unit-erythrocyte (BFU-E). Colonies consisting of erythroid and myeloid cells were scored as CFU-MIX.

Detection of cell surface markers by flow cytometry. A list of antibodies used can be found in Fig.S16. Cells were resuspended at $1x10^6$ cells/50µL in FACS buffer and incubated with FcR blocking reagent (#564219, BD Biosciences) for ten minutes on ice. Next the staining antibody solution was added in 50 µL per 10^6 cells and incubated on ice for 30 minutes in the dark. After incubation, cells were washed once in FACS buffer. For secondary staining, the staining step was repeated with a secondary staining solution. After the wash, cells were resuspended in FACS buffer and analyzed using a LSRII flow cytometer. Debris was excluded using a forward scatter-area and sideward scatter-area gate. Single cells were then gated using a forward scatter-height and forward scatter-width gate. Flow cytometry data were analyzed using FlowJo (version 10.0.8, FlowJo, LLC). **Gating of GFP-positive $CD34^+/CD45RA^-/CD90^+$ cells.** The following antibodies were used. CD34APC (563 Clone): Cat# 561209 from BD; CD90 PE (5e10 Clone): Cat # 328110 from BioLegend; CD45RA APCH7 (5H9 Clone): Cat# 561212 from BDCD45NHP BV421 (D0581283 Clone): Cat# 561291 from BD. Examples for gating $CD34^+/CD45RA^+/CD90^+$ cells as well as bone marrow lineage-positive cells are shown in Fig.S3D.

Detection of human γ-globin expression by intracellular staining. The FIX & PERM™ cell permeabilization kit (#GAS004, Thermo Fisher Scientific) was used and the manufacture's protocol was followed. Briefly, ~$5x10^6$ cells or 8µL whole blood was resuspended in 100µL PBS. 100µL of reagent A (fixation medium) was added and incubated for 2-3 minutes at room temperature. 1mL pre-cooled absolute methanol was then added, mixed, and incubated on ice in the dark for 10 minutes. The samples were then pelleted and washed with FACS buffer (PBS with 1% heat-inactivated FBS), resuspended in 100µL reagent B (permeabilization medium) with 0.6µg hemoglobin γ antibody (Clone 51-7, # sc-21756 PE) (Santa Cruz Biotechnology, Dallas, TX), and incubated for 30 minutes at room temperature. After wash, cells were resuspended in FACS buffer with NucRed™ Live 647 ReadyProbes™ Reagent (#R37106, Thermo Fisher Scientific) and analyzed using a BD LSR II Flow Cytometer (BD Biosciences, San Jose, CA).

Globin HPLC: Individual globin chain levels were quantified on a Shimadzu Prominence instrument with a SPD-10AV diode array detector and an LC-10AT binary pump (Shimadzu, Kyoto, Japan). Vydac 214TP™ C4 Reversed-Phase columns for polypeptides (214TP54 Column, C4, 300 Å, 5 µm, 4.6 mm i.d. x 250 mm) (Hichrom, UK) were used. A 40%-60% gradient mixture of 0.1% trifluoroacetic acid in water/acetonitrile was applied at a rate of 1 mL/min.

Measurement of vector copy number: Total DNA from bone marrow cells, PBMCs or 10-20mg tissue was extracted using the Quick-DNA miniprep kit (Zymo Research). Viral DNA extracted from HDAd-short LCR-γ-globin/mgmt virus was serially diluted and used for a standard curve. qPCR was conducted in triplicate using the power SYBR™ Green PCR master mix on a StepOnePlus real-time PCR system (Applied Biosystems). 9.6 ng DNA (9600 pg/6 pg/cell = ~1600 cells) was used for a 10 µL reaction. For studies in NHP#1, we used human γ -globin primers; for NHP#2, #3, #4 – we used human $mgmt^{P140K}$ primers: human HBG: F: tggccaaacatacattgctaag, R: cccaaatgtttcaattgttcct; Human mgmt: F: tgagaggcaatcctgtcaag, R: CAACCGGTGGCCTTCATGGG.

Vector copy number per single cell/colony: Purified $CD34^+$ cells were plated in ColonyGEL 1402 medium (ReachBio, Seattle, WA) at ~2000 cells/plate, 15 days later, well-isolated colonies were aspirated carefully with a pipette tip and washed with of phosphate-buffered saline. Cell pellets were incubated with 10 µl proteinase K (ThermoFisher) in lysis buffer (50mM KCl, 50mM Tris-HCl (pH8.0), 2.5mM EDTA, 0.45% NP-40, 0.45% Tween-20) at 55°C overnight, followed by 10 min at 95 °C. Samples were diluted to 100-200 µl and 4.8 µl DNA was used in a 10 µl reaction.

Real-time reverse transcription PCR: Total RNA was extracted from $5x10^6$ cells or 100µl blood by using TrIzol™ reagent (Thermo Fisher Scientific) following the manufacture's phenol-chloroform extraction method. Quantitect reverse transcription kit (Qiagen) and power SYBR™ green PCR master mix (Thermo Fisher Scientific) were used. Real time quantitative PCR was performed on a StepOnePlus real-time PCR

system (AB Applied Biosystems). The following primer pairs were used: mouse RPL10 (house-keeping) forward, 5'-TGAAGACATGGTTGCTGAGAAG-3', and reverse, 5'-GAACGATTTGGTAGGGTATAGGAG-3'; human γ-globin forward, aatgtgctggtgaccgtttt-3', and reverse, 5'- agctctgaatcatgggcaaga-3'; mouse α globin forward, 5'- CTGGGGAAGACAAAAGCAAC -3', and reverse, 5'- GCCGTGGCTTACATCAAAGT -3.; Rhesus macaque HBA forward, 5'-cggtcaacttcaagctcctg-3', and reverse, 5'- cggtatttggaggtcagcac-3'; Rhesus macaque GAPDH forward, 5'- atgttcgtcatgggtgtgaa-3', and reverse, 5'- gtcttctgggtggcagtgat-3'; human mgmt^{p140k} forward, 5'- tgagaggcaatcctgtcaag-3', and reverse, 5'- CAACCGGTGGCCTTCATGGG-3'.

Cytometric Bead Array: The NHP Th1/Th2 cytokine CBA kit (BD Biosciences 557800) was used to measure serum levels for IL-2, IL-4, IL-5, IL-6, TNF, IFNγ.

Anti-transgene product antibody ELISA: Plates were coated with recombinant proteins (0.3μg per well in 0.1M Na-bicarbonate buffer pH9.6, o/n at 4°C); human MGMT (Prospectbio), human HBG1 (lsbio), Flp3 (MyBioSource), and SB100x. Serum serial dilutions (starting at 1:50, 3x subsequent dilutions) were added for 1 hour. After washing, HRP-conjugated secondary antibodies against NHP IgG or IgM (Invitrogen PA1-84631 and 62-6820 respectively) were plated at 1:10,000 for 1 hour. After wash, Thermo 1-Step Ultra TMB Solution (ThermoFisher Scientific) was added, and the color was allowed to develop for 7 minutes before stopping with 2N sulfuric acid. Absorbance readings at 450 nm were taken with a plate reader. Antibody reactivity curves were plotted with Graphpad Prism with a 4-parameter curve, and EC50 values were accordingly calculated.

Integration site analysis. Amplification of genomic DNA junctions was performed by linear amplification-mediated PCR and bioinformatic analysis of integration sites was performed as described previously [5].

To analyze the sequencing data, sample specific barcoded sequencing reads were demultiplexed using CASAVA, an Illumina software package. The quality of sequencing runs of resulting fastq files was

evaluated using FastQC (http://www.bioinformatics.babraham.ac.uk/projects/fastqc). Reads starting with the barcode 5’-GTATGTAAACTTCCGACTTCAA-3’ that follows the TA dinucleotide, which is characteristic of SB integration, were aligned against the latest version of rhesus reference genome, using Bowtie2 [7]. Only reads that mapped exactly to a unique position in the reference genome were kept for further analysis, with the threshold of at least 3 uniquely mapped reads per locus. To analyze the distribution of integrations, annotations of exons and CDS of the corresponding reference genome were downloaded and the percentage of integration sites overlapping with the given genomic coordinates were analyzed using BEDTools [8]. Chromosomal distributions of integration sites were visualized on an ideogram using the NCBI Genome Decoration Page (http://www.ncbi.nlm.nih.gov/genome/tools/gdp/).

RNA-Seq analysis: RNA-Seq analysis was performed by Omega Bioservices (Norcross, Georgia, USA). Data were analyzed by Rosalind (https://rosalind.onramp.bio/), with a HyperScale architecture developed by OnRamp BioInformatics, Inc. Reads were trimmed using cutadapt. Quality scores were assessed using FastQC. Individual sample reads were quantified using HTseq4 and normalized via relative log expression using DESeq2 R library. DESeq2 was also used to calculate fold changes and P values and perform optional covariate correction. Enrichment was calculated relative to a set of background genes relevant for the experiment. The volcano plot was generated with custom a Python script that plots log-scale fold change versus P values, and only genes meeting significance $P < 0.01$ are displayed. RNA-Seq data were deposited into the National Center for Biotechnology Information’s Gene Expression Omnibus database (GSE155843).

Statistical analyses*:* Data are presented as means ± standard error of the mean (SEM). For comparisons of multiple groups, one-way and two-way analysis of variance (ANOVA) with Bonferroni post-testing for multiple comparisons was employed. Differences between groups for one grouping variable were

determined by the unpaired, two-tailed Student's t-test. For non-parametric analyses, the Kruskal-Wallis test was used. Statistical analysis was performed using GraphPad Prism version 6.01 (GraphPad Software Inc., La Jolla, CA). *p≤0.05, ** p≤0.0001. A P value less than 0.05 was considered significant.

References:

1. Wang, H., Georgakopoulou, A., Li, C., Liu, Z., Gil, S., Bashyam, A., Yannaki, E., Anagnostopoulos, A., Pande, A., Izsvak, Z., Papayannopoulou, T., et al. (2020). Curative in vivo hematopoietic stem cell gene therapy of murine thalassemia using large regulatory elements. JCI Insight *5*. 10.1172/jci.insight.139538.
2. Wang, H., Georgakopoulou, A., Psatha, N., Li, C., Capsali, C., Samal, H.B., Anagnostopoulos, A., Ehrhardt, A., Izsvak, Z., Papayannopoulou, T., Yannaki, E., et al. (2019). In vivo hematopoietic stem cell gene therapy ameliorates murine thalassemia intermedia. J Clin Invest *129*, 598-615. 10.1172/JCI122836.
3. Wang, H., Richter, M., Psatha, N., Li, C., Kim, J., Liu, J., Ehrhardt, A., Nilsson, S.K., Cao, B., Palmer, D., Ng, P., et al. (2018). A Combined In Vivo HSC Transduction/Selection Approach Results in Efficient and Stable Gene Expression in Peripheral Blood Cells in Mice. Mol Ther Methods Clin Dev *8*, 52-64. 10.1016/j.omtm.2017.11.004.
4. Palmer, D., and Ng, P. (2003). Improved system for helper-dependent adenoviral vector production. Mol Ther *8*, 846-852. S1525001603002855 [pii].
5. Richter, M., Saydaminova, K., Yumul, R., Krishnan, R., Liu, J., Nagy, E.E., Singh, M., Izsvak, Z., Cattaneo, R., Uckert, W., Palmer, D., et al. (2016). In vivo transduction of primitive mobilized hematopoietic stem cells after intravenous injection of integrating adenovirus vectors. Blood *128*, 2206-2217. 10.1182/blood-2016-04-711580.
6. Neildez-Nguyen, T.M., Wajcman, H., Marden, M.C., Bensidhoum, M., Moncollin, V., Giarratana, M.C., Kobari, L., Thierry, D., and Douay, L. (2002). Human erythroid cells produced ex vivo at large scale differentiate into red blood cells in vivo. Nat Biotechnol *20*, 467-472. 10.1038/nbt0502-467.
7. Langmead, B., and Salzberg, S.L. (2012). Fast gapped-read alignment with Bowtie 2. Nat Methods *9*, 357-359. 10.1038/nmeth.1923.
8. Quinlan, A.R., and Hall, I.M. (2010). BEDTools: a flexible suite of utilities for comparing genomic features. Bioinformatics *26*, 841-842. 10.1093/bioinformatics/btq033.

Accession #___21-112_
Submission Date 12 Jul 2021

University of Washington
National Primate Research Center

DIAGNOSTIC LABORATORY BIOPSY REPORT

Requester___________AG____ Investigator___AL_____ Animal ID #___A20141
Species_______Mm____ Requester's Phone________________

Date of Death__12 Jul 21_ Date of Necropsy 12 Jul 21__Time__9:30am Pathologist ____AB____

Nutritional Condition: ☐ Adequate ☒ Marginal ☐ Poor ☐ Obese

Other Tests Required: ☐ Sero ☐ Micro ☐ Parasit ☐ Other_________________

Other Diagnostic Samples __________________

Type of report: ☒ Final 30 Jul 2021 ☒ Preliminary __12 July 2021_______ ☐ Amended _________

Clinical History: this animal was assigned to the "Veevo NHP Study with long LCR" protocol. There is history of chronic weight loss with muscle wasting and a body condition score of 1. There is mild anemia and thrombocytosis with unremarkable chemistry panels.

Gross Description: a female rhesus macaque, 8 years, 5.27 kg, is submitted for examination after blood and bone marrow collection, euthanasia and saline perfusion. There is minimal dental tartar and moderate muscle wasting along the back and legs. There are scant adipose tissue stores, both with the subcutis and internally. The stomach contains scant brown mucous material, the small intestinal is mildly thickened and stool with the colon is slightly soft. The lungs are mildly congested and edematous. Other organ systems are grossly unremarkable.

Gross Diagnosis(es):

Probable gastroenterocolitis, with adipose tissue loss and muscle wasting.
Pulmonary congestion and edema.

Gross Comments: tissues were collected per protocol.

Histological Findings:

Lungs: there is multifocal to diffuse, mild to moderate mixed interstitial pneumonitis with alveolar edema and fibrin. One section contains an area of severe alveolar edema, interstitial pneumonia, with respiratory epithelial hyperplasia and moderate numbers of alveolar macrophages and interstitial mixed inflammatory cells. The pleura appears unremarkable. No organisms are seen.

Liver: multiple sections are examined, with similar findings. There is multifocal periportal to random or centrilobular, mild to moderate chronic cholangiohepatitis / hepatitis, with mild centrilobular hepatocellular

atrophy. Few individual necrotic hepatocytes are seen, associated with mild mixed inflammation. Vessels and the capsular surface are unremarkable.

Sections from the cerebrum, brainstem, cerebellum, spleen (diffuse, mild lymphoid hyperplasia), gall bladder, kidneys (bilateral, multifocal, moderate membranous glomerulonephritis with interstitial fibrosis and mild chronic interstitial nephritis), heart, lymph nodes (diffuse, moderate lymphoid hyperplasia), pancreas (diffuse, moderate interstitial edema with mild, multifocal chronic pancreatitis), adrenal glands, thyroid glands, trachea, esophagus, aorta, skin with mammary gland tissue, skeletal muscle (right thigh), tongue, uterus, ovary/oviduct, urinary bladder (focal mild chronic cystitis), and gastrointestinal tract (diffuse, moderate to severe, lymphocytic, plasmacytic and mildly eosinophilic gastroenterocolitis with moderate to marked submucosal and mesenteria lymphoid hyperplasia) are examined with with exceptions of stated changes, appear unremarkable.

Final Principal Diagnosis(es):

1. Pneumonitis, multifocal to coalescing, moderate to focally severe, mixed, with alveolar edema and histiocytosis; lungs.
2. Cholangiohepatitis, mild to moderate with mild centrilobular hepatocellular atrophy and rare single cell necrosis; liver.
3. Gastroenterocolitis, diffuse, moderate to severe, chronic and eosinophilic, with lymphoid hyperplasia.
4. Splenic lymphoid hyperplasia, diffuse, mild.
5. Mild chronic pancreatitis.
6. Membranous glomerulonephritis, moderate, with mild to moderate interstitial fibrosis and chronic nephritis; bilateral kidneys.

Histology Comments: microscopic examination is consistent with moderate to sevre inflammation within the gastrointestinal tract, with mild pancreatitis and cholangiohepatitis / hepatitis, supportive of chronic weight loss. Gastroenterocolitis is commonly seen and may be multifactorial in etiology.

Pneumonitis is multifocal, with areas of more diffuse inflammation and alveolar edema. Such changes may be seen after total body irradiation – interpret in conjunction with experimental manipulations. No organisms are seen within the lung sections.

The kidney lesions are mild to moderate and likely subclinical at this stage.

Pathologist______AB__________
